# Molecular targets and strategies in the development of nucleic acid cancer vaccines: from shared to personalized antigens

**DOI:** 10.1186/s12929-024-01082-x

**Published:** 2024-10-09

**Authors:** Wei-Yu Chi, Yingying Hu, Hsin-Che Huang, Hui-Hsuan Kuo, Shu-Hong Lin, Chun-Tien Jimmy Kuo, Julia Tao, Darrell Fan, Yi-Min Huang, Annie A. Wu, Chien-Fu Hung, T.-C. Wu

**Affiliations:** 1 Physiology, Biophysics and Systems Biology Graduate Program, Weill Cornell Medicine, New York, NY USA; 2 Tri-Institutional PhD Program in Chemical Biology, Memorial Sloan Kettering Cancer Center, New York, NY USA; 3 Pharmacology PhD Program, Weill Cornell Medicine, New York, NY USA; 4 Department of Epidemiology, The University of Texas MD Anderson Cancer Center, Houston, TX USA; 5 The University of Texas Graduate School of Biomedical Sciences at Houston and MD Anderson Cancer Center, Houston, TX USA; 6 Division of Pharmaceutics and Pharmacology, College of Pharmacy, The Ohio State University, Columbus, OH USA; 7 Department of Pathology, Johns Hopkins School of Medicine, 1550 Orleans St, CRB II Room 309, Baltimore, MD 21287 USA; 8 Department of Oncology, Johns Hopkins School of Medicine, Baltimore, MD USA; 9 Department of Obstetrics and Gynecology, Johns Hopkins School of Medicine, Baltimore, MD USA; 10 Department of Molecular Microbiology and Immunology, Bloomberg School of Public Health, Johns Hopkins School of Medicine, Baltimore, MD USA

**Keywords:** Cancer vaccine, Tumor antigens, Neoantigens, DNA, mRNA, Clinical trial

## Abstract

Recent breakthroughs in cancer immunotherapies have emphasized the importance of harnessing the immune system for treating cancer. Vaccines, which have traditionally been used to promote protective immunity against pathogens, are now being explored as a method to target cancer neoantigens. Over the past few years, extensive preclinical research and more than a hundred clinical trials have been dedicated to investigating various approaches to neoantigen discovery and vaccine formulations, encouraging development of personalized medicine. Nucleic acids (DNA and mRNA) have become particularly promising platform for the development of these cancer immunotherapies. This shift towards nucleic acid-based personalized vaccines has been facilitated by advancements in molecular techniques for identifying neoantigens, antigen prediction methodologies, and the development of new vaccine platforms. Generating these personalized vaccines involves a comprehensive pipeline that includes sequencing of patient tumor samples, data analysis for antigen prediction, and tailored vaccine manufacturing. In this review, we will discuss the various shared and personalized antigens used for cancer vaccine development and introduce strategies for identifying neoantigens through the characterization of gene mutation, transcription, translation and post translational modifications associated with oncogenesis. In addition, we will focus on the most up-to-date nucleic acid vaccine platforms, discuss the limitations of cancer vaccines as well as provide potential solutions, and raise key clinical and technical considerations in vaccine development.

**Supplementary Information:**

The online version contains supplementary material available at 10.1186/s12929-024-01082-x.

## Introduction

Cancer vaccines have become of great interest in the development of cancer therapy due to their capacity to leverage immune cells against cancer, specifically targeting malignant cells while preserving healthy ones. To design an effective vaccine enabling a precise immune response, it is crucial to select the appropriate target antigen [1]. Neoantigens stand out as potential targets for the immune system. These antigens are derived from abnormal proteins generated from somatic mutations or mutation-independent processes present in cancer cells, thus they are expressed specifically in cancer cells but are not present in normal tissues. Cancer vaccines targeting neoantigens can be used in conjunction with immune checkpoint inhibitors (ICIs), a class of drugs blocking proteins that downregulate CD8 T cell’s cytotoxicity. ICIs have demonstrated significant efficacy against cancer types with high mutational burden [2, 3]. However, not every neoantigen adequately induces a functional T-cell response. Lang et al. have proposed a classification of neoantigens into three groups based on their immunogenicity [4]: guarding neoantigens, which are strongly immunogenic and rapidly eliminated; restrained neoantigens, which are only recognized by T cells unleashed by ICIs; and ignored neoantigens, which are not immunogenic even with ICI therapy. Ignored neoantigens are presented on MHC class I complexes, yet fail to effectively prime naive T cells. It is hypothesized that vaccination may enhance T cell responses to these ignored neoantigens, thereby augmenting therapeutic antitumor effects.

Due to their patient-specific nature, neoantigens are particularly applicable in the field of personalized medicine. There are currently more than 100 active or completed clinical trials that can be found on clinicaltrials.gov when searching for the terms “neoantigens” and “vaccines”. While a clear benefit has not been demonstrated in a majority of these therapeutic vaccine trials, two recent trials— mRNA-4157 in melanoma (NCT03897881) [5] and autogene cevumeran (BNT122, RO7198457) in pancreatic cancer (NCT04161755) [6]—have reported evidence of clinical benefit, as indicated by prolonged recurrence-free survival (RFS). These successes can be attributed to significant advancement in personalized strategies for cancer treatment [7]. For example, advances in molecular techniques such as next-generation sequencing (NGS) technologies have facilitated detailed examination of the cancer genome, including genetic alterations, gene expression, and epigenetic modifications. Analysis of a patient’s genomic information can provide a personalized approach to identifying clinically relevant target antigens and selecting individually appropriate treatment methods.

**Fig. 1:**
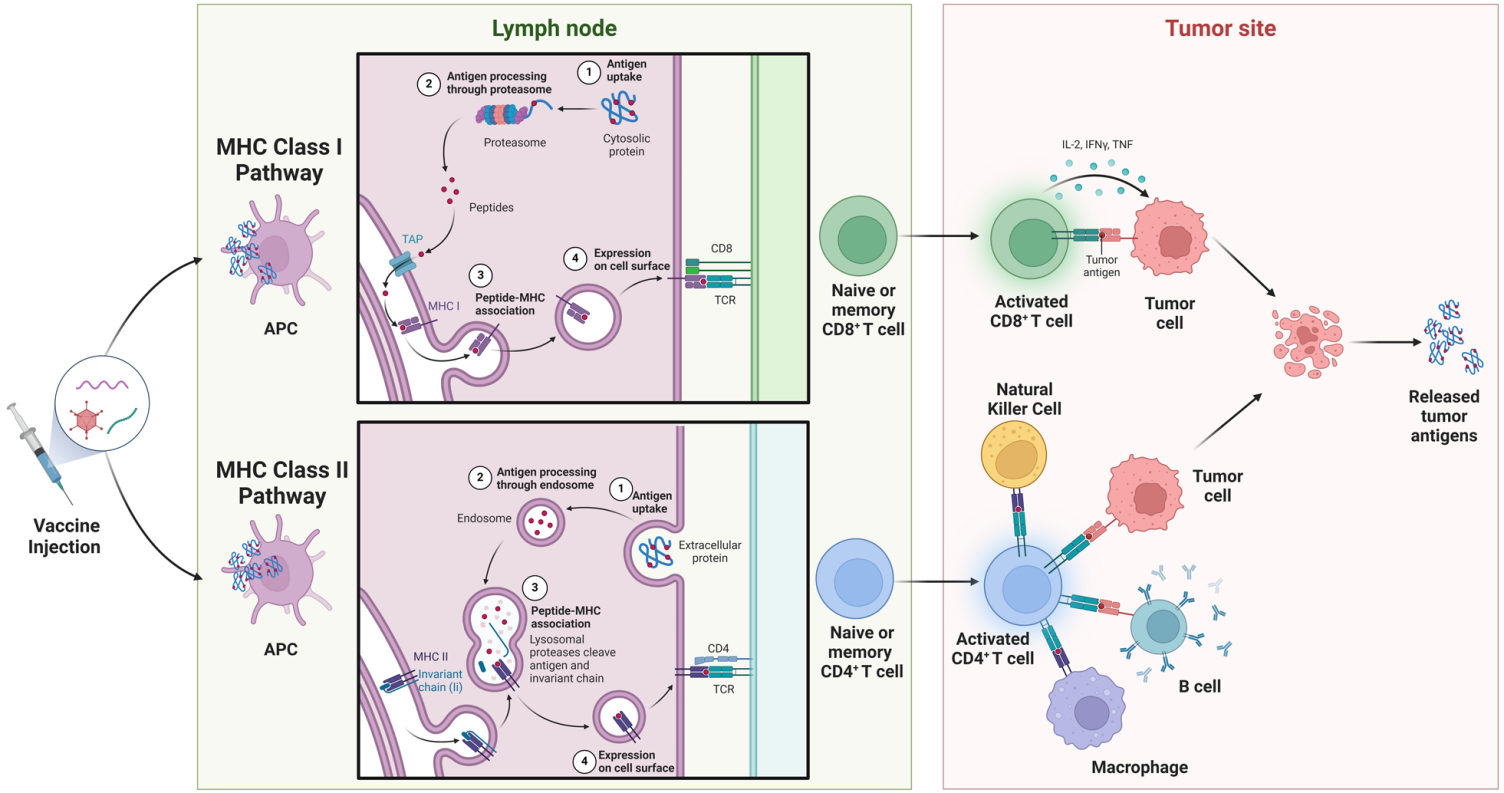
MHC class I and II antigen processing pathways. The endogenous pathway features MHC class I mediated antigen processing. Through MHC class I, cellular proteins are processed into peptide fragments by proteasomes and aminopeptidases, which are then transported by TAP into the endoplasmic reticulum to be loaded onto MHC class I molecules. These peptide-MHC-I complexes are then displayed on the cellular surface for recognition by CD8 T cells, activating cytotoxic T cells for the killing of virus-infected or cancerous cells. The exogenous pathway features MHC class II-mediated antigen processing. Through MHC class II, exogenous antigens are endocytosed by APCs, processed in endosomal and lysosomal compartments, and then loaded onto MHC class II molecules. These peptide-MHC-II complexes are then displayed on the cellular surface for recognition by CD4 T cells, triggering various immune responses such as cytokine secretions

Beyond identifying antigens, developing cancer vaccines also requires the choice of an appropriate platform for effective delivery. An ideal cancer vaccine design should include highly immunogenic neoantigens that robustly induce both helper and cytotoxic T-cell responses (Fig. 1). Mouse studies have shown that vaccines incorporating both CD4 and CD8-specific neoantigens are more effective than those only containing CD8 antigens, demonstrating the importance of including both MHC class I and class II antigens in cancer vaccine design [8]. Current options for delivering neoantigens include dendritic cell-based, microbial vector-based, tumor cell-based, peptide-based, DNA-based, and mRNA-based vaccine platforms. Among these, the nucleic acid-based vaccine platforms have recently gained increased interest due to vaccine development efforts during the COVID-19 pandemic. Not only were various mRNA vaccines approved for human use, but a DNA vaccine in humans (ZyCoV-D) was also approved for the first time [9]. Furthermore, both DNA and mRNA vaccines are known for their ease of preparation, rapid adaptability for clinical use, and excellent safety profiles, although efforts are still needed to improve delivery efficiency [10].

With these principles in mind, this review will focus on therapeutic nucleic acid-based cancer vaccines designed to enhance antigen presentation, thereby promoting specific immune cell recognition of tumors as a cancer treatment strategy. We will provide an overview of the following topics: 1. Types of cancer antigens, including shared and personalized antigens and their use in cancer vaccines. 2. Identifying neoantigens and optimizing selection of the highest quality and most immunogenic candidates for personalized cancer vaccination. 3. Vaccine platforms available for delivery of the antigen to generate a lasting immune response, particularly focusing on DNA and mRNA vaccines. 4. Discussions on the limitations of cancer vaccines and potential solutions. 5. Clinical considerations of personalized cancer vaccines as adjuvants in early-stage cancers and as maintenance treatments in advanced, metastatic cancers.

## Types of cancer antigens

Tumor antigens can be categorized into shared or personalized antigens by their expression frequency [11]. Shared antigens are relatively common across different patients and could provide a promising off-the-shelf immunotherapy option. Recent developments in the field have also given rise to vaccines targeting personalized antigens, which are more specific to individual patients and could induce higher immune response. Here, we discuss different types of antigens and their respective clinical development in cancer vaccines.

### Shared cancer antigens

Shared cancer antigens, including tumor-associated antigens (TAAs) and tumor-specific antigens (TSAs), were previously identified and characterized for their role in cancer. TAAs are overexpressed in cancer cells relative to normal cells, whereas TSAs are exclusively found in cancer cells. These properties make TAAs and TSAs potent targets for immunotherapeutic strategies [12]. Ideal therapeutic antigens are characterized by their therapeutic function, immunogenicity, role of antigen in oncogenicity, specificity, expression level of antigen-positive cells, number of targetable antigenic epitopes, and cellular location of antigen expression [13]. These antigens are essential in developing vaccines aimed at boosting the immune system's ability to recognize and eliminate cancer cells expressing these markers.

### Tumor-associated antigens (TAAs)

TAAs, which are overexpressed in cancer cells relative to normal cells, are commonly chosen antigens in vaccine development. Among TAAs, cancer-testis antigens (CTAs) are a specialized subset that are thought to be highly immunogenic due to their restricted expression in immune privileged sites, such as the testis, and their absence in lymphoid tissues [14]. NY-ESO-1 is a CTA expressed across various malignancies, including neuroblastoma, myeloma, bladder cancer, non-small cell lung cancer (NSCLC), ovarian cancer, prostate cancer, and breast cancer, to name a few [14]. Cancer vaccines targeting NY-ESO-1, such as pPJV7611 [15], SCIB2 [16], CV9202 [17], and BNT111 [18], are being developed for the treatment of solid cancers such as NSCLC and melanoma. MAGEs are another example of CTAs, and are among the first TAAs identified at the molecular level [19]. Several members of the MAGE family have been targeted by cancer vaccine candidates. These include BNT111 targeting MAGE-A3 [18], CV9202 targeting MAGE-C1 and MAGE-C2 [17], and a DNA vaccine candidate targeting MAGE-A1 and MAGE-A3 (NCT04049864).

Human telomerase reverse transcriptase (hTERT), a protein involved in telomere synthesis, is an important TAA highly expressed in cancers including hepatocellular carcinoma, melanoma, bladder cancer, and glioblastoma [20]. The hTERT antigen is targeted by cancer vaccines such as GV-1001 [21, 22], INO-1400, INO-1401 [23], INO-5401[24], and INVAC-1 (IVS-2001) [25]. These vaccines combine hTERT with other antigens or modified hTERT protein to target solid tumors or chronic lymphocytic leukemia. One example, GV-1001, in combination with chemotherapy agents gemcitabine/capecitabine showed improved overall survival in serum eotaxin-high patients with untreated advanced pancreatic ductal adenocarcinoma (PDAC), and has been approved in South Korea [22]. Human epidermal growth factor receptor (EGFR) family members are also common targets due to their high expression in cancers including breast cancers and NSCLC. Vaccines targeting the EGFR family include AST301 (pNGVL3-hICD) [26] and AVX901 (VRP-HER2) [27] targeting HER2, and pING-hHER3FL targeting HER3 in solid cancers (NCT03832855). Prostate-specific antigen (PSA), prostate specific membrane antigen (PSMA), prostate acid phosphatase (PAP), and prostate stem cell antigen (PSCA) are antigens highly expressed in prostate cancers, and have been developed as targets for prostate cancer vaccines, such as BNT112 (PRO-MERIT) [28] (NCT04382898), and PROSTVAC-VF [29, 30]. One key milestone is the FDA approval of Sipuleucel-T, a personalized DC vaccine that pulses patient dendritic cells with PAP and GM-CSF ex vivo before reinfusion [31]. This FDA approval followed three Phase 3 trials: D9901 [32], D9901/D9902A [33], and D9902B (IMPACT) [34]. Insulin-like growth factor binding protein 2 (IGFBP2) is often highly expressed in breast or ovarian cancer, and has also been developed as a cancer vaccine target in AST-201 [35] and WOKVAC [36, 37]. Additional File 1: Table 1 and Table 2 provide a comprehensive list of mRNA and DNA vaccines targeting TAAs.

### Viral tumor-specific antigens (TSAs)

Viral TSAs are found in cancers associated with viral infections, including human papillomavirus (HPV), the Epstein–Barr virus (EBV), and hepatitis B virus (HBV). These viruses cause around 13% of all cancers [38]. HPV oncoproteins E6 and E7 are known to interfere with cell cycle proliferation [39, 40], leading to malignancies such as cervical, oropharyngeal, or anal cancers [41]. These oncoproteins are critical in vaccines targeting HPV-related cancers. Vaccines targeting HPV-associated malignancies include VB10.16 [42], VGX-3100 [43], GX-188E [44], and pNGVL4aCRTE6E7L2 [45], pNGVL4a Sig/E7(detox)/HSP70 (PVX-2)/pBI-11WT1 [46], BNT113 [47], and pBI-11 [48]. EBV was the first virus found to cause human cancer, and it has been associated with various lymphomas and epithelial cancers [49]. Currently, WGc-043 developed by Westgene targets EBV-associated antigens for EBV + cancer treatment (NCT05714748). Another mRNA vaccine candidate targeting hepatitis B virus (HBV) for hepatocellular carcinoma is also under early development (NCT05738447). Additional File 1: Table 3 and Table 4 provide a comprehensive list of mRNA and DNA vaccines targeting TSAs.

### Non-viral tumor-specific antigens (public neoantigens)

Non-viral TSAs often encompass patient-specific neoantigens arising from mutations in driver genes, such as those found in Kirsten rat sarcoma virus (KRAS), tumor protein p53 (TP53), or Wilm’s tumor suppressor gene 1 (WT1). KRAS has a high mutation rate in PDAC, NSCLC, and colorectal cancer (CRC) [50]. Vaccine candidates targeting KRAS mutations include mRNA-5671 (V491), which targets KRAS G12D, G12V, G13D or G12C mutations (NCT03948763), and another mRNA vaccine candidate targeting G12C, G12D, or G12V (NCT05202561). SLATE-001, a mRNA-LNP vaccine developed by Gritstone Bio, targets multiple TSAs including TP53 and KRAS that were identified using Gritstone’s EDGE neoantigen discovery platform [51]. INO-5401, developed by Inovio Pharmaceuticals, targets the TSA WT1 along with hTERT and PSMA to treat urothelial carcinoma and glioblastoma (NCT03502785, NCT03491683) [24].

Despite the promise of shared cancer antigens in vaccine development, several challenges persist. Shared antigens like TAAs are often present at low levels in normal cells [52], but there is still the potential for off-tumor, on-target toxicity and autoimmune responses [12]. In addition, clinical trials of TAA-based vaccines have shown limited efficacy, leading to the discontinuation of several candidates [53]. For example, Moderna’s mRNA-2416, targeting OX40L, was prematurely halted due to insufficient efficacy (NCT03323398). Similarly, CureVac’s CV9202 was discontinued after showing a low overall response rate of 3.8% [17]. On the other hand, targeting a single shared TSA might not be an effective therapeutic strategy. CDX-110 (rindopepimut), a peptide-based vaccine developed by Pfizer and Celldex to target EGFRvIII—a TSA commonly expressed in glioblastoma, breast cancer, and lung cancer [54, 55]—was discontinued after phase III trials failed to meet efficacy endpoints [56]. These challenges underscore the necessity for identifying multiple antigens that can elicit stronger immune responses, leading to the development of vaccines tailored to individual immunogenic profiles.

### Personalized neoantigens (private neoantigens)

Personalized cancer vaccines target unique antigens derived from an individual's tumor mutations (mutation-dependent neoantigens) and non-mutation-based processes (mutation-independent neoantigens), offering novel targets for the immune system. Earlier attempts at vaccine development involved using inactivated whole tumor cell lysate as vaccines in an autologous or allogeneic manner [57]. For example, M-Vax comprises of autologous melanoma cells modified with dinitrophenyl (DNP) combined with Bacille Calmette-Guerin (BCG) to boost its immunogenicity to the same individual from which the vaccine was prepared [58]. Melacine used allogeneic melanoma cell lysates combined with DETOX, an immunologic adjuvant to provide immunity to patients other than the patient from whom the melanoma cells were derived [59]. Both vaccines were approved in the 2000s but are currently unavailable due to suboptimal efficacy and better alternatives [60]. Currently, by specifically focusing on neoantigens unique to each patient's tumor, personalized vaccines aim to generate a robust and targeted immune reaction, potentially improving the efficacy of cancer immunotherapy. Additional File 1: Table 5 and Table 6 provide a comprehensive list of mRNA and DNA vaccines targeting personalized neoantigens.

### Mutation-dependent neoantigens

Mutation-dependent neoantigens can arise from single nucleotide variations (SNVs), small insertions and deletions (indels) and structural variants (SVs) [61]. SNV-derived neoantigens are the most studied mutation type as they are easier to detect. High SNV frequency is a biomarker for high tumor mutation burden (TMB) and correlates with higher response rates to ICI therapy, particularly in melanoma and NSCLC [62–65]. However, SNV-type neoantigens are not as immunogenic due to the high similarity to unmutated proteins [66, 67]. In contrast, structural variations, which are changes > 50 base pairs (bp) in length resulting from insertion, deletion, duplications, inversions, or translocations, can cause frameshifts and gene fusions. These alterations lead to dramatic differences in amino acid sequences that can give rise to highly specific and immunogenic neoantigens. Their lack of normal protein function targets them as defective products for rapid destruction and presentation on MHC class I molecules [68]. For example, indels or frameshift mutations, which create novel open reading frames and produce abnormal proteins, are shown to generate nine times more high-affinity neoantigen binders than SNVs [69]. Tumor infiltrating lymphocytes (TILs) in clear cell renal cell carcinoma recognize both SNVs and frameshift-derived neoantigens [70]. Gene fusions are also demonstrated to produce neoantigens that contribute to immune surveillance [71]. It was estimated that gene fusions lead to sixfold more neoantigens than SNVs and indels [72]. Lastly, endogenous retroelements can be reactivated and give rise to neoantigens that are otherwise not expressed in normal tissues [12, 73].

While these mutation-dependent neoantigens have become increasingly easier to identify due to the extensive genetic information provided through NGS sequencing, there are a number of limitations to this approach. For instance, these vaccines against mutation-dependent neoantigens often produce an inefficient immune response, potentially due to low expression levels of the neoantigen, ineffective presentation due to low neoantigen stability, or immunosuppressive tumor microenvironment [74–76]. Furthermore, intra-tumoral heterogeneity and clonal diversity pose challenges for identifying neoantigens that are universally expressed across all tumor cells, potentially leading to immune escape and tumor progression [77].

### Mutation-independent neoantigens

While mutation-dependent strategies for neoantigens have been extensively studied, the low mutational burden in many cancers limits the potential of mutation-dependent neoantigen vaccine strategies. Thus, it is crucial to explore mutation-independent neoantigens. One angle that has gained particular attention is the “dark proteome” [78]. This term describes the alternative protein products produced from non-canonical, also known as “cryptic,” alterations in transcription and translation, constituting the so-called “dark” portion of the proteome [79]. These can arise from alternative splicing [80], usage of non-AUG start codons [81, 82], ribosomal frameshift errors [83, 84], readthrough events into the 3’-untranslated region [85, 86], and expression from non-canonical ORF-containing genes [87–89]. Post-translational products may also be targeted; in particular, cancer-specific pathways may lead to expression of unique cryptic neoantigens through alterations of the post-translational modification patterns [90]. Furthermore, proteasomal peptide splicing can generate non-canonical MHC class I ligands to elicit tumor-specific cytotoxic lymphocyte (CTL) responses [91, 92].

Similar to mutation-dependent neoantigens, many cryptic protein products’ short half-lives enable their rapid degradation and efficient presentation on MHC class I molecules [93], making them desirable for the generation of novel cancer vaccines based on mutation-independent neoantigen targets. However, despite the potential of targeting genetically diverse tumors with shared mutation-independent neoantigens, these neoantigens are often non-essential for tumor survival and may be expressed heterogeneously, making them susceptible to immune editing and reduced vaccine effectiveness. Thus, the “dark proteome” displays much potential, but further validation is still required to determine its effectiveness in cancer immunotherapy.

### Notable personalized cancer vaccine trials

Recent advances in personalized cancer vaccines have focused on identifying tumor-specific mutations to produce neoantigen vaccines for individual patients. Consequently, cancers with high TMB are particularly favorable for cancer vaccine development [94–96]. Several vaccines are currently in clinical development, with leading initiatives by companies such as Moderna, Merck, BioNTech, and Genentech progressing into phase II or III clinical trials.

mRNA-4157 (V940), a lipid nanoparticle (LNP)-based mRNA cancer vaccine developed by Moderna and Merk, is designed to target up to 34 individualized neoantigens [5]. In phase II trials, it has shown promise against resected stages III-IV melanoma when used in conjunction with pembrolizumab, showing extended RFS and distant metastasis-free survival (DMFS) compared to pembrolizumab alone [97]. The treatment regimen involves administering nine intramuscular doses at 3-week intervals. From the initial sample collection to the delivery of the encapsulated mRNA, the process takes about 6 weeks, with ongoing efforts to reduce this timeframe to 30 days. Phase III trials are actively proceeding for both melanoma and NSCLC [98] (NCT05933577, NCT06077760, NCT03313778).

Autogene cevumeran (BNT122 or RO7198457) is an mRNA cancer vaccine developed by BioNTech and Genentech. The vaccine is formulated to encode up to 20 personalized neoantigens [6, 99]. In a phase Ib trial with atezolizumab, the vaccine induced neoantigen-specific T-cell responses in 77% of patients, with mostly mild side effects including fatigue and nausea [100]. Autogene cevumeran is currently in Phase II clinical trials for resected PDAC, aiming to evaluate its safety and efficacy compared to standard-of-care treatments, with endpoints including disease-free survival (DFS) and overall survival (NCT05968326).

GNOS-PVO2 is a DNA cancer vaccine developed by Geneos Therapeutics for advanced hepatocellular carcinoma (HCC) [101]. It includes a DNA plasmid encoding up to 40 neoantigens, as well as a second DNA plasmid encoding cytokine interleukin-12 (IL-12) as an adjuvant. The vaccine is delivered via intradermal injection followed by electroporation. It is currently in phase I/IIa trials in combination with pembrolizumab (NCT04251117) and has been shown to generate a localized response to expressed antigens at the site of injection in patients with advanced HCC. The objective response rate was 30.6%, with 8.3% achieving complete responses, linked to the number of neoantigens included. The vaccine induced significant neoantigen-specific T cell responses in 86.4% of evaluable patients, leading to the activation, proliferation, and infiltration of vaccine-specific CD4 and CD8 T cells into tumors.

There are additional personalized cancer vaccines under early-stage development or phase I clinical trials. VB10.NEO, co-developed by Nykode and Genentech, is a DNA-based vaccine targeting up to 20 neoantigens in phase I/IIa trials of locally advanced or metastatic solid tumors [102]. EVX-02 is a DNA-based vaccine targeting up to 13 antigens for melanoma, currently under phase I/IIa trials (NCT04455503). Nouscom’s NOUS-PEV utilizes a gorilla adenovirus [9] prime and a modified vaccinia Ankara boost to target up to 60 neoantigens in a phase I trial for metastatic NSCLC and melanoma [103, 104]. GRANITE (ZVexNeo) is a heterologous mRNA-based vaccine in phase I trial developed by Gritstone Bio that includes a chimpanzee adenovirus vector (ChAd) and a self-amplifying mRNA encoding 20 personalized neoantigens [105]. Transgene developed a MVA-based vaccine, TG4050, targeting up to 30 neoantigens in phase I trials for head and neck cancers and ovarian cancers [1, 106]. iNeo-Vac-P01 from Hangzhou Neoantigen Therapeutics focuses on peptides targeting 5–20 neoantigens in a phase I trial for pancreatic cancers [107]. Lastly, vaccine candidates from University of Florida utilizes whole-tumor-derived mRNA and the multi-lamellar RNA lipid particle aggregates (LPAs) technique to generate mRNA-based vaccines [108, 109]. These diverse approaches represent the forefront of personalized cancer vaccine development with various neoantigen targets and lead indications across different phases of clinical trials.

## Neoantigen identification

As the demand for discovering antigens with stronger immune responses increases, identifying actionable novel neoantigens becomes crucial in developing personalized cancer vaccines.

Current methods for identifying tumor neoantigens involve three main steps: target identification, neoantigen prediction, and target validation. Upon validation, the shortlisted antigens are formulated into vaccines using various platforms.

To identify specific neoantigens for cancer vaccines, whether mutation-dependent or mutation-independent, one may consider the steps in antigen generation, processing and presentation. These steps include: (1) transcription of genes to mRNA, (2) translation of mRNA to proteins, (3) proteasomal degradation of proteins to peptides, (4) loading on to MHC molecules, (5) peptide-MHC molecules recognition by T cell receptors (TCRs), and (6) activation of T cells to elicit immune responses (Fig. 1) [66]. Therefore, each step of cellular antigen generation and presentation requires corresponding computational algorithms or models to address and predict neoantigens [66, 110]. These tools help filter through all possible neoantigens to generate a manageable shortlist for subsequent validation.

Here, we will first cover the strategies for identifying neoantigens derived from tumor mutations and follow with identification of mutation independent neoantigens. We will then detail the process of optimizing neoantigen immunogenicity through various predictive processes.

### Mutation-dependent neoantigen identification from sequencing data

The first step in identifying mutation-dependent neoantigens is to find tumor-specific mutations. This can be done by algorithms or variant callers that identify genetic variants by comparing the DNA: targeted panels, whole exome sequencing [111], or whole genome sequencing (WGS). RNA sequencing (RNA-seq) can also be conducted utilizing patients’ tumor samples and matched normal samples, typically from peripheral blood mononuclear cells (PBMC) [112]. Each method faces a specific set of challenge: (1) tumor samples are usually contaminated with normal tissues, (2) mutations are usually heterozygous, (3) tumor-specific mutations are mostly subclonal, i.e. each mutation is usually found in a subset of the cancer cells in the tumor samples, and (4) quality tissue samples are of low availability.

Extensive efforts have been taken to compare the accuracy of variant callers for SNVs and indels as well as structural variants. Bohnert et al. compared the performance of 13 exome callers in detecting somatic SNVs in 100% pure tumor samples [113]. They found that 80.7% of all known SNVs were identified by all callers, with six callers achieving a sensitivity above 90% and five callers achieving a precision above 90%. However, the performance of variant callers decreased significantly with lower tumor purity. When testing with 90% admixture data, the seven best callers detected only 36–55% SNVs. A similar decline in performance was observed with varying levels of admixture when the callers lack normal reference, and the top-performing callers in tumor-normal pair settings did not perform best in tumor-only data. The challenge of impure tumor tissue sequencing could be addressed by adjusting for tumor purity and allowing tumor-only variant calls [112]. In a more recent effort, Pei et al. assessed the performance of variant callers for detecting both SNVs and indels, finding over 90% precision when tumor purity is above 10%, which demonstrates the significant improvements in variant callers over the years [114]. Structural variant callers were also evaluated in detecting germline mutations [115], showing that size of structural variant greatly impacts detection. Several callers achieved over 80% sensitivity for detecting events larger than 10,000 bp, while sensitivity for 1,000 bp events were 60% or lower. Additional benchmarking studies are needed to better evaluate the performance of somatic structural variant callers.

Many clinically available tumor tissues are preserved as formalin-fixed and paraffin-embedded (FFPE) blocks, which are valuable sources for large-scale genomic studies. Despite excellent preservation of tissue morphology and stabilization of biomolecules, the FFPE procedure can create nucleotide modifications that result in sequencing artifacts. Ruiz et al. analyzed EGFR and KRAS in 47 lung cancer samples using PCR and Sanger sequencing and found that DNA amplification occurred in only 50% of the FFPE samples compared to 100% in fresh frozen tissues [116]. These issues were subsequently ameliorated by both experimental and computational strategies, e.g. filtering out artifacts specific for FFPE tissues [112, 117, 118]. In practice, ensembles of multiple callers often achieve results better than any single caller, and thus are often adopted in academic and industrial research settings [119]. Another trend in the field is the development of neural network-based variant callers, whose performances are comparable to ensemble methods [120].

RNA sequencing (RNA-seq) provides both quantitative insights into gene expression and qualitative information on sequence changes, making it advantageous over DNA sequencing to study TAAs and TSAs. One significant advantage of RNA-seq is the flexibility in aligning sequencing reads either to the reference genome with gap-aware aligners or to the reference transcriptome using traditional aligners [112]. While aligning to the reference transcriptome is computationally straightforward, aligning to the reference genome enables the detection of novel alternative splice variants that may be tumor-specific. However, limitations persist in using RNA-seq to identify SNVs. Quinn et al. tested several strategies to call germline variants using GATK and SAMtools, finding that sensitivity and specificity were higher than 85% for variants with 10X or higher coverage [121]. Zhao et al. demonstrated that read length significantly influences SNV detection performance, with a 125 bp read length resulting in a 34.5% false positive rate, which decreased to 6.1% with 150 bp reads [122].

As single-cell RNA-seq has become more accessible, researchers have evaluated its potential for variant identification. Liu et al. compared variants discovered in single-cell and bulk RNA-seq and reported that the median sensitivities of SNVs called in single-cell context ranged from 63.4 to 82.6%, with true positive rates exceeding 90% for several callers tested [123]. These studies demonstrated consistency between single-cell and bulk RNA variant calls in germline settings. However, more studies are needed to better account for potential issues inherent to single cell RNA-seq.

Protein expression data from large-scale proteomics studies, such as the Clinical Proteomic Tumor Analysis Consortium (CPTAC), can also be incorporated into the neoantigen identification pipeline [124]. CPTAC integrates comprehensive genomic and proteomic data, including WGS, WES, regular proteome, and phosphoproteomes, allowing researchers to bridge the gap between DNA, RNA, and protein expression levels and prioritize neoantigens for effective vaccine design.

### Mutation-independent neoantigen identification from sequencing data

To comprehensively identify tumor-specific neoantigens that are independent of genetic mutations, advanced techniques leveraging high-throughput sequencing data are essential. One such approach is ribosome profiling (RiboSeq), which captures ribosome-protected mRNA fragments to enable global mapping of the translatome, or the translated protein products in tumor and normal tissue [125, 126]. This provides insights into the active translation landscape of cancer cells, enabling the detection of translated protein products beyond those encoded by mutated genes. In conjunction with RiboSeq, immunopeptidomics emerges as a pivotal methodology for neoantigen discovery. Utilizing mass spectrometry, immunopeptidomics enables the direct identification of antigenic peptides presented by MHC class I molecules on the surface of cancer cells [88, 127]. This technique offers a unique advantage by directly interrogating the repertoire of peptides presented by cancer cells, thus bypassing the need for predictive algorithms based solely on genomic or transcriptomic data. By integrating RiboSeq with immunopeptidomics, researchers can identify a spectrum of tumor-specific cryptic translation products that may serve as neoantigens.

### MHC genotyping

The genotyping of human MHC, also known as human leukocyte antigen (HLA), determines the MHC alleles present in an individual. This process is important for optimizing presentation of both mutation-dependent and mutation-independent neoantigens. Studies have shown that different MHC alleles have their preferred peptide sequences [128]. Therefore, MHC genotyping is important to accurately predict the peptide antigens presented on the cell surface. Several tools exist to determine MHC alleles from WGS, WES or RNA-seq **(**Additional File 2: Supplementary Table 1). For MHC class I genotyping, Optitype offers good specificity and selectivity compared to other tools [129, 130]. There are tools that offer genotyping of both MHC class I and class II from NGS sequencing data, such as seq2HLA [131], ArcasHLA [132], and ATHLATES [133] (Additional File 2: Supplementary Table 1). However, current NGS sequencing methods often fail to capture the complete sequence of MHC alleles due to shallow read depth or short-read sequencing setups. Practically, targeted probe capture based clinical tests and Sanger sequencing are preferred [134].

### MHC-peptide binding prediction

The presentation of any given antigen on the MHC molecule is a highly selective process influenced by protein preferences, including proteasome degradation of protein, transporter associated with antigen processing protein (TAP), and MHC loading. Research has demonstrated that specific positions within 8–11 mer peptides are critical for MHC class I binding [135]. Notably, positions 2 and 9 in 9-mer peptides serve as anchor positions, often favoring hydrophobic amino acids across most HLA genotypes [136].

Over the years, various methods have been developed for predicting MHC binding, as summarized in Additional File 2: Supplementary Table 2. Early tools relied on scoring functions and position-weighted matrices, while more recent approaches employ neural networks. The evolution of MHC binding prediction tools has been highly dependent on data, particularly on our understanding of antigen binding data and MHC alleles. Earlier tools were limited by sparse antigen affinity data and a small subset of MHC alleles. However, recent advances have provided more extensive MS peptide elution data (peptidome or ligandome) and immunogenicity data, including rare MHC alleles [137]. As a result, the predictive accuracy of these tools has improved significantly over time.

Several tools are available for predicting MHC class I binding in limited MHC alleles or in pan-alleles (Additional File 2: Supplementary Table 2). For example, NetMHC is a neural network-based tool to predict peptide binding to a limited selection of MHC class I alleles, trained on antigens from the Immune Epitope Database (IEDB), which primarily includes viral epitopes for common MHC alleles [138]. Conversely, NetMHCpan is a pan-specific model for MHC class I alleles, integrating binding affinity data with mass spectrometry peptidome data [139, 140]. In recent years, deep learning models have gained popularity. For example, Gritstone bio’s EDGE model uses deep learning neural networks to identify tumor neoantigens from tumor MHC class I peptides via mass spectrometry [141]. BigMHC, an ensemble neural network model trained on MS peptide elute data and assays of antigen-specific immune response, claims to be superior to other state-of-the-art tools [142].

Predicting binding specificity for MHC class II is even more challenging than for MHC class I. MHC class I typically binds shorter peptides (8–11 amino acids), whereas MHC class II binds longer peptides (13–25 amino acids) with greater length variability. This is because the peptide-binding groove of MHC class I has closed ends, while that of MHC class II has open ends, allowing for more diverse peptide sequences and binding to different MHC class II molecules [143, 144]. Additionally, MHC class II can form heterodimers, adding to its diversity and expanding the search space compared to MHC class I [145, 146]. Given the plethora of MHC binding prediction tools, the IEDB Analysis Resource regularly updates the performance of common predictors through weekly automated benchmarks. As of April 2024, the IEDB recommends neural network-based prediction models NetMHCpan 4.1 and NetMHCIIpan 4.1 [147].

### TCR tools & immunogenic prediction

A challenging puzzle in predicting peptide immunogenicity is TCR binding prediction. Once MHC presents the peptide, TCR recognizes the MHC-peptide complex (pMHC) and activates the T cell. A TCR contains two chains: an alpha chain and a beta chain. On each chain there are three regions specific for recognizing pMHC: the complementarity-determining regions (CDRs) CDR1, CDR2, and CDR3. CDR1 and CDR2 recognize mainly the MHC, while CDR3 is responsible for recognizing the MHC-bound peptide [148]. Therefore, as opposed to MHC-peptide binding prediction, TCR binding prediction is extremely challenging because there are four components: MHC, peptide, CDR3β, and CDR3α.

Several tools predict the binding of TCR and pMHC **(**Additional File 2: Supplementary Table 3). Because CDR3β was initially considered more predictive for binding, earlier tools were predominantly trained on CDR3β alone. Later tools, however, were trained on both CDR3α and CDR3β, with some also incorporating CDR1, CDR2, and additional data. Earlier tools also implemented score-based, distance-based, or simple machine learning prediction, while later tools implemented a wide array of deep learning neural networks to predict the binding.

Most of the tools were trained on pMHC-TCR binding data from public databases including VDJdb [149], McPAS-TCR [150], Immune Epitope Database (IEDB) [151], TBAdb [152], and ImmuneCODE^™^ [153]. Some recent tools also took advantage of datasets from NGS-based assays, such as 10X Genomics Single Cell Immune Profiling [154, 155]. These single-cell assays allow for higher throughput acquisition of pMHC-TCR binding data [156].

Similar to peptide MHC binding, there is a benchmark dataset to compare between different tools [157]. Overall, most methods have comparable performances across the benchmark dataset, but they do not generalize well to unseen datasets [158, 159]. This is mostly due to the scarce dataset compared to the vast search space of all possible combinations of pMHC and TCR [158]. Newer technologies such as 10 × single-cell TCR sequencing efforts can generate large amounts of data in a single study and would be suitable for future work [160].

### Clonality

One important predictor of neoantigen immunogenicity is clonality. Neoantigens can be clonal or subclonal based on their expression pattern within the tumor. A clonal neoantigen is expressed across all cancer cells in the tumor, while a subclonal neoantigen is only expressed in a portion of the tumor tissue. Studies have shown that clonal neoantigens are a better predictor of response to immunotherapies [161]. Thus, clonal neoantigens are more likely to be the target of cancer vaccines and immunotherapies. However, some clonal driver mutations might be harder to target due to selection of antigens eliciting immune escape [4, 162]. This is likely the reason behind the failure of some vaccines or therapeutics targeting shared antigens (e.g. EGFRvIII) in clinical trials [163, 164]. Personalized cancer vaccines can address this problem by including a wide range of neoantigens across clonal and subclonal neoantigens [4]. There are some tools used to estimate clonality using bulk sequencing data [165], including PyClone [166], FastClone [167] and SciClone [168]. To better resolve neoantigen clonality, single-cell sequencing can be used to pinpoint cell populations that harbor clonal or subclonal mutations. Ongoing developments in spatial transcriptomics could further complement single cell sequencing and reveal functional states of tumor cells in relation to their location and interaction with tumor microenvironment, especially immune cells.

### Dissimilarity

There might be thousands of neo-antigenic peptides identified from a patient. To shortlist the candidates to a handful of peptides that are manageable and can be validated using cell assays, we need to consider dissimilarity. The importance of dissimilarity to self-antigen is two-fold: it prevents possible vaccine-induced autoimmunity and increases immunogenicity.

To prevent possible vaccine-induced autoimmunity, the neoantigens are usually compared with the human proteome or HLA ligandome from normal tissue for similarity. These databases include Uniprot [169], Ensembl [170], GENCODE [171], IEDB [147], and HLA Ligand Atlas [172]. Usually, the neoantigen candidates that match or are highly similar to normal human HLA ligands are removed or subject to experimental validation.

There are also several endeavors to predict immunogenicity using dissimilarity to self-antigen. For example, Luksza et al. generated a neoantigen fitness model to rank neoantigens and predict tumor response to immunotherapy [173]. Richman et al. included both dissimilarity to self-antigens and homology to pathogens in their model [174]. These studies have been shown to link neoantigen quality to peptide immunogenicity and clinical outcomes.

### Antigen selection and prioritization

With many different tools at hand, each tool predicts neoantigens with the highest score in terms of MHC binding or TCR binding. One challenge lies in integrating all data processing pipelines to select and prioritize neoantigens down to a feasible number for validation. Several open source pipelines such as OpenVax or pVACtools have been published [175, 176]. In general, most pipelines use a rank-based or score-based method to filter and prioritize neoantigens [177, 178], although the actual parameters set for each application are usually empirical or proprietary.

### Validation of vaccine candidates

Despite advancements in computational neoantigen predictions, a significant proportion of predicted neoantigens fail to elicit T-cell responses. Therefore, functional assays are crucial to validate selection methods and assess the immunogenicity of the selected neoantigens. These assays include tests to confirm TCR recognition, activation, and cytotoxicity, as well as efforts to capture the T-cell repertoire for vaccine validations. Here, we discuss several common assays used to evaluate T-cell properties.

It is important to develop a strategy to validate and prioritize the identified neoantigens for the development of cancer vaccines. To achieve this, T cells are taken from the patient, either derived from their PBMCs or TILs. These cells can then be co-cultured with neoantigen-loaded APCs [179, 180], and CD8 + and/or CD4 + T-cell activation can be measured through detection of the cytokine IFN-γ using enzyme-linked immunosorbent spot assay (ELISpot) [181, 182]**.** This can also be done using intracellular cytokine staining followed by flow cytometry analysis. Positive results indicate that T cells recognize and respond to the neoantigen peptides.

Complementary to T cell activation or TCR recognition assays, cytotoxicity assays assess T-cell killing capabilities [182–184]. TILs can be isolated from tumor samples via fluorescence-activated cell sorting (FACS), expanded, and tested for their ability to recognize and kill cancer cells expressing the target neoantigens. This method provides direct evidence of T-cell recognition and response to neoantigens within the tumor microenvironment.

In vivo models, such as mouse models or humanized mouse models, can also be used to validate the immunogenicity and therapeutic efficacy of neoantigens [185, 186]. These models involve assessment of the immune response and tumor growth in the mice in response to neoantigen vaccination or adoptive T-cell transfer. However, such an approach is not practical to be incorporated in the validation of the neoantigen vaccination for human usage, due to the time and resources involved.

While the above assays confirm TCR recognition and activity, they do not provide a comprehensive view of the T cell repertoire. Therefore, methods have been developed for unbiased understanding of T cell diversity and neoantigen response. Traditional methods like Sanger sequencing and multiparameter flow cytometry have low sensitivity. High-throughput NGS and targeted TCR sequencing offer better insights into T cell diversity and neoantigen response [187, 188]. Single-cell RNA-seq or TCR-seq techniques further enhance our understanding of the number of T cells recognizing specific epitopes and the overall T cell diversity [189, 190]. The mutation-related neoantigen-specific function extension (MANAFEST) analysis is also a sensitive platform to determine presence of neoantigen-specific T cells through antigenic peptide stimulation and TCR sequencing [191]. Taken together, these functional assays are pivotal in validating computational predictions and ensuring the effectiveness of neoantigen-based cancer vaccines. However, the assays are relatively time-consuming and labor intensive, therefore the development of high-throughput and unbiased computational strategies is essential [66].

Each of the aforementioned validation strategies rely on pre-existing T cell immunity in patients to validate neoantigenic targets. However, a drawback to this is the potential to miss other important neoantigenic epitopes expressed by tumor cells. These neoantigen peptides may be expressed at low levels or presented minimally by MHC molecules on tumor and/or antigen-presenting cells, yet they could still be immunogenic when combined with potent adjuvants. In addition, effector CTLs, once fully activated and differentiated, have a lower activation threshold and require fewer peptide-MHC class I complexes for killing. However, to effectively prime naive T cells, high densities of these complexes are required, which may be insufficient in tumors [192]. Furthermore, the immunosuppressive tumor microenvironment (TME) may inhibit antigen presentation [193, 194]. Initial priming also often relies on cross-presentation; however, some neoantigens may not cross-present effectively and thus fail to elicit spontaneous immunity [75]. This is due to tumor mutations often leading to unstable protein products which are rapidly digested by proteasomes and directly presented on MHC class I molecules in tumor cells five times more efficiently than canonical proteins [93]. Consequently, when unstable antigens are poorly cross-presented, CTLs may not be primed efficiently [74, 195]. A potent vaccine along with adjuvants may be able to overcome this challenge [196]. Altogether, neoantigen peptides can serve as excellent targets for tumor eradication, so efforts should be taken to ensure that these epitopes are not overlooked.

## Vaccine platforms

**Fig. 2:**
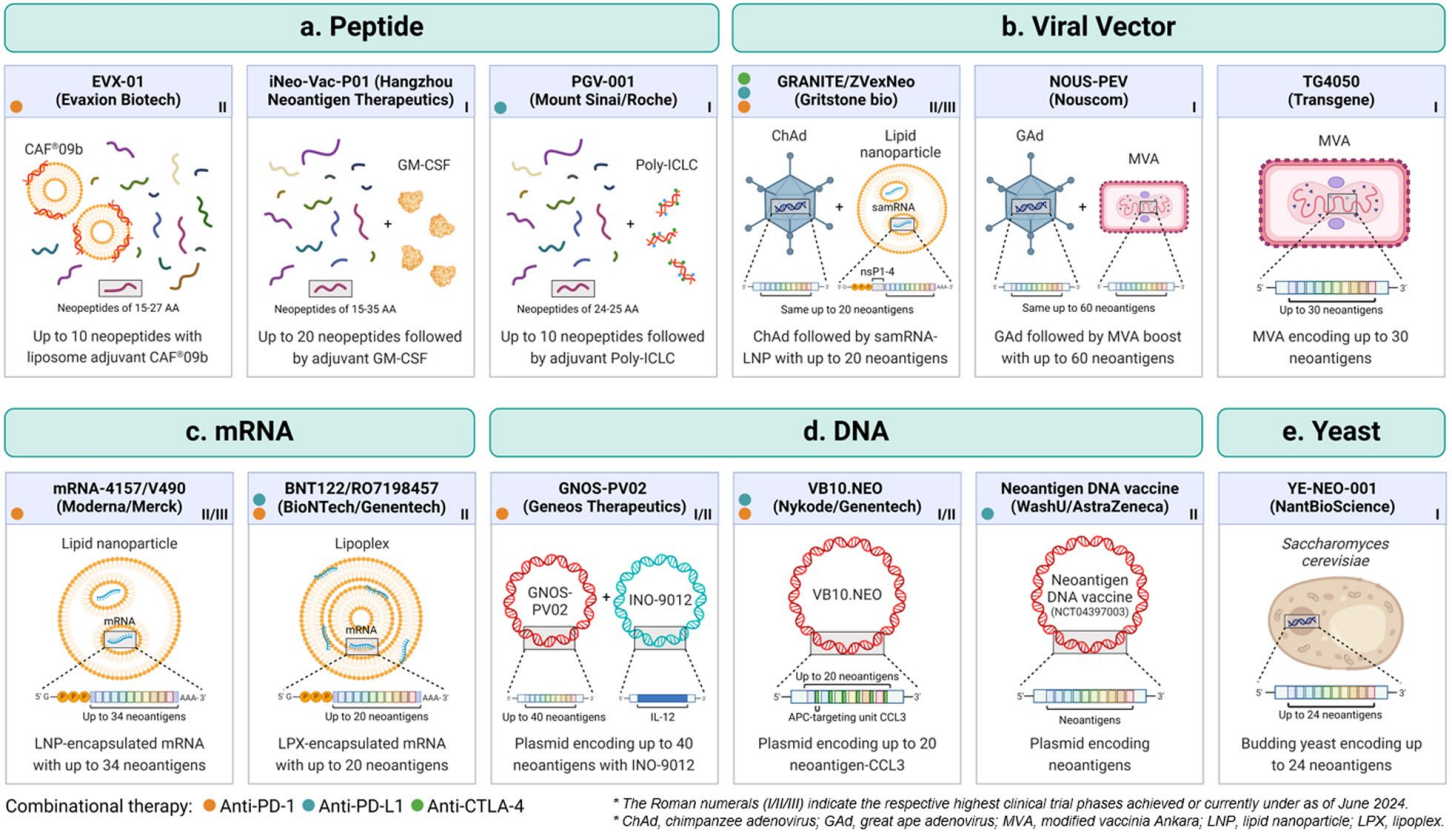
Personalized cancer vaccine platforms: relevant clinical trials. Various cancer vaccine platforms have been utilized to deliver neoantigens for cancer immunotherapeutic response, typically targeting APCs to enhance T cell activation. This figure details some relevant clinical trials for each of the selected types. **a** Peptide vaccines directly deliver neoantigenic peptides for activation of immune response. These were frequently employed in early cancer vaccine developments due to their safety, ease of design, and ability to directly exploit the MHC presentation system. Limitations to this system include low stability and immunogenicity, high cost, and potential insolubility or aggregation. **b** Viral vector vaccines utilize defective or attenuated viruses lacking replicative genes to deliver neoantigen genes into host cells. Advantages include efficient delivery and strong and prolonged immune responses. However, a disadvantage is that the intrinsic immunogenicity to the vector itself may limit efficacy with repeated doses. **c** mRNA vaccines are delivered into the host cell cytoplasm, translated to protein by ribosomes, and processed for MHC presentation. RNA vaccines have recently gained attention for their flexibility and versatility in design, ease of production, and safety. However, they are less stable than DNA vaccines. **d** DNA vaccines must be delivered into the nucleus to be transcribed to mRNA, translated to protein, and processed for presentation. Thus, concerns of DNA vaccines include low transfection efficiency and immunogenicity, and low but potential risk of integration into the genome. However, they are also more stable and long-lasting than RNA vaccines, flexibly designed, and safe. **e** Yeast-based vaccines utilize genetically engineered yeast cells to express neoantigen peptides or proteins, which are released inside APCs once they are phagocytosed. They have gained attention in recent years for their non-pathogenicity, inherent adjuvant nature, and ease of production. However, a limitation is their different glycosylation pattern compared to human glycosylation

Several vaccine platforms have been used for the development of cancer vaccines including peptide-based vaccines, vector-based vaccines, mRNA-based vaccines and DNA-based vaccines (Fig. 2). Along with favorable safety profiles, peptide vaccines were frequently employed in earlier cancer vaccine developments because of the mature peptide synthesis methodology [197]. Peptide vaccines also have favorable safety profiles. While MHC-presented peptides are short (8–12 amino acids) in length, it has been shown that immunizing a long peptide encompassing around 30 amino acids of the antigen more effectively induces sustained effector CD8 T cell reactivity than the minimal peptide [198]. It is likely because a long peptide ensures the antigen is uptaken and processed by APCs, rather than being loaded onto MHC directly, thereby inducing a comprehensive immune response [198]. Therefore, synthetic long peptides (SLPs), typically 25–35 amino acids in length containing multiple epitopes or longer segments of the target proteins, have become a common design strategy in peptide-based cancer vaccines. However, one limitation of peptide vaccines is their suboptimal immunogenicity, which necessitates the inclusion of immunostimulatory adjuvants such as toll-like receptor (TLR) agonists. These adjuvants are critical for initiating the innate immune response, robust T cell activation, and effective processing against virus infections or cancer cells [199]. In addition, due to the variable chemical properties of amino acids, some peptide sequences can be less soluble or prone to aggregate, posing potential challenges in vaccine manufacturing [197].

Vector-based vaccines such as viral, bacterial and yeast vectors have also been widely used in gene therapy and vaccine delivery due to their high potency and ability to accommodate specific genetic sequences for therapeutic or antigenic purposes. Commonly employed viral vectors include defective or attenuated derivatives of adenovirus, poxviruses, and alphaviruses [200]. These vectors are engineered to lack essential viral genes necessary for replication, enhancing their safety as delivery platforms [200]. Viral vectors have established a robust safety profile and efficacy in various applications, evidenced by their use in approved vaccines for COVID-19 and Ebola [200]. However, their intrinsic immunogenicity is a double-edged sword. While capable of eliciting strong innate and adaptive immune responses that are prolonged and durable, the resulting neutralizing antibodies can hinder subsequent re-immunization using the same vector, thereby limiting options for repeated dosing [201]. Additionally, pre-existing immunity to commonly used vectors like adenovirus or measles in some patients can diminish the efficacy of the therapy or the response to booster doses [201]. To address these challenges, viral vectors can be employed in initial vaccination regimes, with subsequent (booster) doses delivered via other vector types [202]. Alternatively, viral vectors engineered from other species, such as chimpanzee and great ape adenoviral vectors, can be used to circumvent immunity issues and enhance the overall effectiveness of the vaccination strategy [105, 203]. New avenues of cancer vaccine delivery have also been on the rise, including yeast vectors. For example, a current phase I clinical trial (NCT03552718) is evaluating the safety and efficacy of the YE-NEO-001 vaccine derived from a heat-killed yeast engineered to express neoantigen peptides, for patients with potentially curatively treated solid cancer. Yeast vectors have inherent adjuvant nature and can be produced rapidly and cost-effectively. One limitation to keep in mind, however, is the significant difference in glycosylation pattern between yeast and human species. Thus, production in yeast must consider optimization for human-like glycosylation [204].

Despite the advancements in peptide- and vector-based vaccines, nucleic acid-based vaccines offer several key advantages. (1) Nucleic acid-based vaccines can engage more efficiently with MHC class I and class II presentation than peptide-based vaccines [205]. (2) Nucleic acid-based vaccines have a simpler, quicker, and cheaper production process than peptide- and vector-based vaccines [206]. This facilitates easier modification to address manufacturing obstacles in the context of personalized vaccines. (3) Multidose vaccination regimens are possible without inducing preexisting immunity as with vector-based vaccines [201]. (4) Nucleic acid-based vaccines contain fewer additives than peptide-based vaccines in the final solution [207, 208]. The following section will focus on delivery strategies and technical considerations to enhance nucleic acid-based vaccine developments.

### mRNA-based vaccines

Research advancements in mRNA therapeutics have surged since the COVID-19 pandemic in 2020, marked by the emergency use of two mRNA vaccines that revolutionized the field of immunization [200, 209]. mRNA vaccines offer flexible sequence design and permit the encoding of tumor antigens and immunomodulating signals to boost both innate and adaptive immune responses [210]. Furthermore, unlike DNA vaccines, mRNA vaccines eliminate risks associated with potential host genome integration and allow for transient, high-level expression of encoded proteins. These vaccines are generated via in vitro transcription and are suitable for rapid large-scale production [211, 212]. mRNA vaccines have demonstrated the potential for a rapid response to emerging infectious diseases by delivering genetic materials through well-tolerated carrier platforms, most notably LNPs and liposomes [213].

Liposomes have been widely used for mRNA vaccine delivery due to their flexibility and modifiability [214]. Recently, a liposomal mRNA delivery system was created by manipulating liposomes with a cholesterol-modified cationic peptide DP7, with the aim of optimizing delivery of neoantigen mRNA to dendritic cells and enhancing the immune adjuvant effect [215]. Another example is the mRNA cancer vaccine autogene cevumeran, developed by BioNTech and Genentech for surgically resected PDAC and currently in phase II clinical trials (NCT04161755, NCT05968326). This vaccine utilizes mRNA-lipoplex nanoparticles for delivery to provide increased stability to the mRNA [6, 216]. Despite these successes, a limitation of lipid vectors is their instability and propensity to aggregate, affecting storability and immune response initiation.

Despite the various delivery strategies listed, RNA vaccines have faced several technical challenges including low translation efficiency and lower stability. Some of these challenges have been addressed in recent years. First, translation efficiency can be enhanced by using codons preferred by eukaryotic ribosomes, optimizing untranslated regions (UTRs), managing GC content, and incorporating nucleotide modifications [217]. Second, the molecular stability of mRNA can be improved by optimizing secondary structures and delivery in carriers such as LNPs [218]. Additional considerations regarding mRNA string design are discussed in “Box: Special Considerations for mRNA-based vaccines”.

**Box: special considerations for mRNA-based vaccines** The mRNA-based vaccines have the additional layer of complexity in string design. A huge amount of effort has been dedicated to disentangle the machinery involved in the translation of mRNA to provide guidance in string design.

RNA acquires secondary structure as it is synthesized. The folding structure of a RNA molecule is the result of interplay among base pairing [219], positively charged ions [220], and RNA binding proteins (RBP) [221]. Structures of manufactured RNA tend to be more predictable because of the control of available RBP or lack thereof. On the other hand, the structure of RNA synthesized within cells can be cell-type dependent due to differing compositions of RBP [221]. Stability of RNA structure can be partially determined using the minimum free energy (MFE) calculation [222], which considers thermodynamic parameters from base pairing and formation of loops, bulges, or other structures. However, most algorithms do not consider the influence of RBP and thus might require additional validation and revision on its structure when RBP is present.

Both primary and secondary structures of RNA contribute to stability and translatability. The composition of nucleotides and sequence of the RNA dictate potential base pairing. As cytosine and guanine pairing results in three hydrogen bonds as opposed to two in the case of adenosine and uridine pairing, RNA molecules with higher GC content could be more structured. The length, location, and strength of palindromic sequences can determine which types of secondary structure are more stable and likely to exist longer for a given RNA molecule. The formation of secondary structure also has an impact on whether parts of the RNA are accessible for RBP [223] and inhibitory nucleotides like miRNA [224]. Generally speaking, more structured RNA has lower translatability and longer half-life [225]. These two characteristics could result in a smoother and longer-lasting dosing curve rather than an expression spike that disappears shortly. Possible mechanisms for the low translatability and longer half-life include the need for ATP-powered unwinding by helicase [226], and the spacing out of ribosomes to avoid RNA decay mechanisms triggered by ribosome stalling or collision [227]. Despite potential contribution of secondary structures and higher GC content, immunity against overly structured RNAs or RNAs with high GC content could be catastrophic [228–230].

Non-canonical nucleotides change the thermodynamic properties of RNA molecules and interaction with RBP. Studies have shown that non-canonical nucleotides could prevent RNA molecules from triggering unwanted immune responses via TLR pathways [231]. The incorporation of modified nucleotides has also been shown to influence secondary structure [232] and RBP interactions [233]. Certain structure predictions of given RNA molecules have incorporated experimentally determined characteristics of modified nucleotides [234, 235]. However, more general predictions of translatability and stability might be warranted.

An mRNA molecule can be divided into the following functional regions: 5’ UTR, coding sequence [236], 3’ UTR, and poly-A tail. Besides overall considerations mentioned in the previous paragraphs, CDS additionally requires codon optimization. The 20 canonical amino acids are encoded by 64 3-nucleotide codons. Overuse of a specific codon could deplete corresponding tRNA leading to slowing down or stalling of translation [237]. One of the major goals in RNA optimization algorithms is to manage usage of each codon while maintaining the desired amino acid sequence. These algorithms often explore different combinations of RNA string sequence to balance relative synonymous codon usage [238–240] or minimize tRNA adaptation index [241] which incorporates translation efficiencies of each codon-anticodon pairing.

### mRNA-loaded dendritic cell-based vaccine

Ex vivo modified dendritic cells, such as those loaded with mRNA encoding tumor antigens, can also be utilized for cancer vaccination. To prepare these DC-based vaccines, monocytes are first isolated from patients and differentiated into DCs in culture with factors such as granulocyte–macrophage colony-stimulating factor (GM-CSF) and interleukin-4 (IL-4) [242]. Then, these DCs can be transfected with mRNA of interest before the entire complex is transfused back into the patient. One example is a Phase 1 trial investigating the use of TLR7/8-matured DCs transfected with mRNA encoding for tumor-associated antigens WT1 and PRAME to treat AML patients (NCT01734304) [243]. Results indicated that this treatment method was feasible, safe, and sufficient to induce an antigen-specific immune response. Another example is a Phase 2 trial for PSA, PAP, survivin, and hTERT mRNA-transfected DCs to treat patients with metastatic castration-resistant prostate cancer. This DC-based cancer vaccine was delivered in conjunction with docetaxel for this patient population and was proven to be safe (NCT01446731) [244]. Additional File 1: Table 7 provides a comprehensive list of mRNA-loaded dendritic cell-based vaccines targeting TAAs, TSAs, and personalized neoantigens.

### DNA-based vaccine

**Fig. 3:**
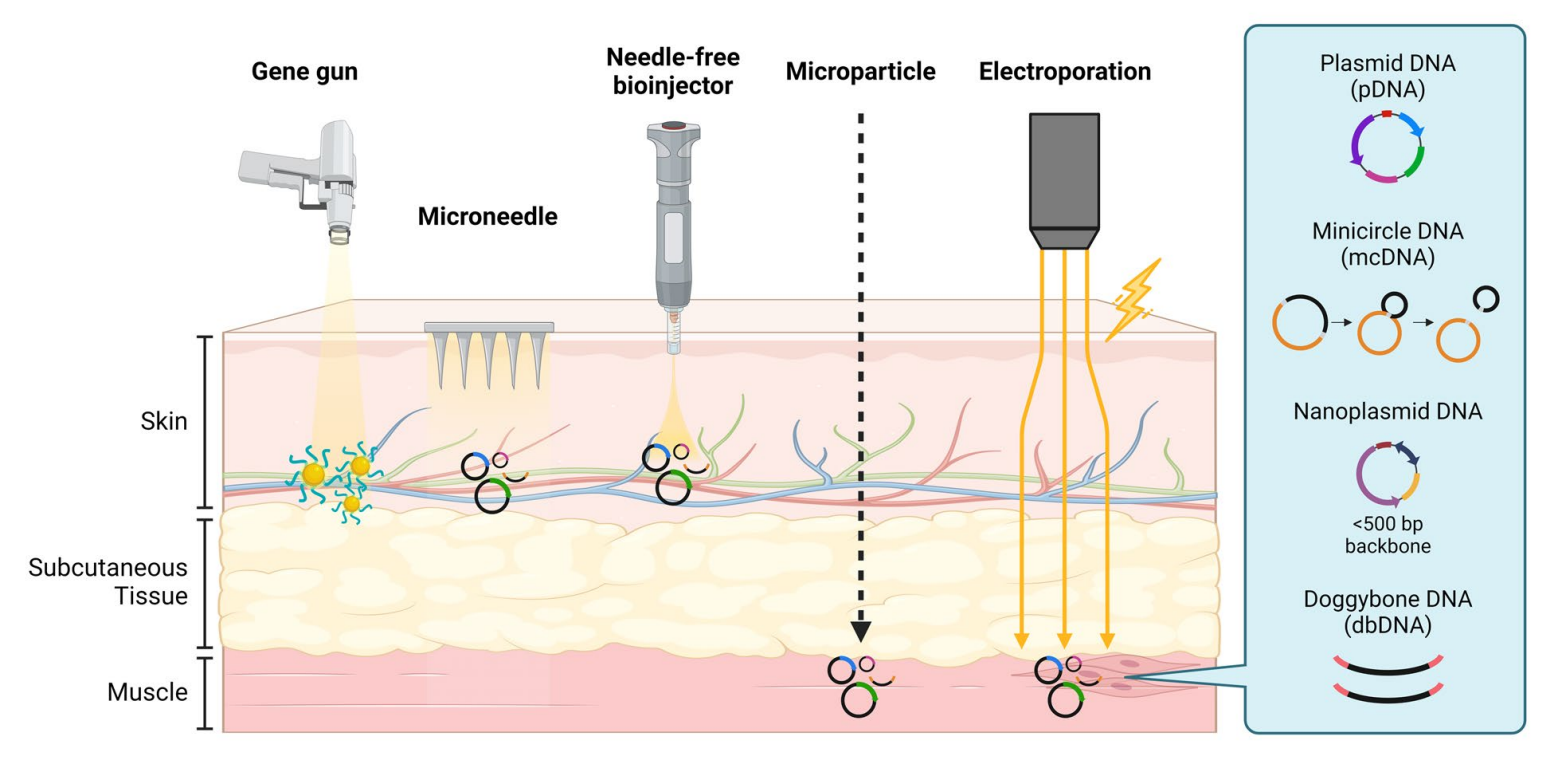
Delivery strategies for the various types of DNA-based constructs. There are various types of DNA vectors for delivery, including plasmid DNA (pDNA), minicircle DNA (mcDNA), nanoplasmid DNA (npDNA), and doggybone DNA (dbDNA). Unlike pDNA, these new forms lack the prokaryotic sequences that induce inflammation and the genes for antibiotic resistance. mcDNA are excised by a recombinase enzyme, leaving only the gene of interest and necessary regulatory elements. npDNA uses antisense RNA for plasmid selection without using antibiotic resistance markers. dbRNA generates a linear, covalently closed DNA without using bacterial sequences. There are five strategies to deliver these DNA constructs. One physical method is using a gene gun, which coats the DNA on heavy metals to be ejected into tissue. Another method is using microneedle, which are tiny needles (< 1 mm) to deliver DNA only to the outermost layer of the skin. This layer is enriched with immune cells such as Langerhan cells, which would facilitate in leading to a potent immune response. A third method is a needle-free biojector, which generates a high-pressure liquid stream to push the DNA into tissue without needles. A fourth method is to use microparticles (1–1000 µm) to encapsulate the DNA for controlled and targeted delivery into specific tissues. The last method is to use electroporation-mediated needle delivery, which uses electric fields to disrupt the membrane transiently. This allows the delivered DNA to enter the cells more efficiently

Currently, less clinical trials for cancer vaccine development have employed DNA vaccines compared to RNA vaccines. DNA must be delivered to the nucleus to be able to be transcribed, and thus needs to overcome both the cell membrane and nuclear envelope [245, 246]. Despite this, DNA has various advantages compared to RNA and overcoming the delivery hurdle may enable its more widespread use. Figure 3 illustrates the various delivery strategies and types of DNA-based constructs for delivery in current vaccine developments.

First, the safety and tolerability of DNA vaccines has been confirmed through many clinical trials [247–249]. Data has demonstrated that the risk of DNA vaccines integrating into the genome is lower than that of spontaneous mutations in the genome [246, 250]. Furthermore, DNA vaccines are much more stable and thus are often delivered as “naked DNA vaccines” without packaging, causing fewer undesirable effects [9, 251, 252]. In contrast, RNA vaccines typically require packaging with biomaterials such as lipid nanoparticles (LNPs) which contain polyethylene glycol (PEG) which may induce serious allergic effects. Without the need for LNPs, this also lowers DNA vaccine production cost compared to RNA vaccines and makes it possible to scale-up production [246, 253]. Another key advantage to DNA vaccines is its long-lasting effect compared to mRNA, as it can persist stably and non-integrated in the cell nucleus for up to 6 months, unlike RNA which is degraded rapidly in the cytoplasm [254].

Various methods have been employed for DNA vaccine delivery. Due to the need to pass through both the cell membrane and enter the nucleus, simple intramuscular (I.M.) injection of naked DNA has the limitation of low transfection efficacy. Thus, DNA vaccines are generally administered through electroporation, which enhances uptake and generates an immunogenic response. Despite the efficacy of DNA vaccines, the specialized equipment required for electroporation techniques poses significant limitations for mass vaccination efforts, as shown during the COVID-19 pandemic [200]. Consequently, research continues into alternative delivery platforms to expand accessibility and application. Other physical methods include the gene gun (DNA coated on heavy metals and ejected with high force into tissue), jet injection (high-pressure liquid stream to penetrate the skin without use of needles), and microneedles (small arrays of micro-sized needles which reach into the epidermis and dermis). These methods enable intradermal (I.D.) delivery, which has been shown to improve the immunogenicity of DNA vaccines compared to the typical I.M. injection. Due to the higher prevalence of APCs in the skin than the muscle, I.D. delivery allows their direct transfection, leading to enhanced antigen presentation [255, 256]. Multiple clinical trials currently employ such methods. For instance, a phase I clinical trial (NCT00199849) employing gene gun delivery of NY-ESO-1 DNA plasmid vaccine (pPJV7611) demonstrated that in 15 patients without antigen-specific immune response, 93% developed CD4 T cell response and 33% developed CD8 T cell responses post-vaccination, although the response was not long-lived, potentially due to regulatory T cell interaction [15]. GNOS-PV02 (NCT04251117), mentioned previously and currently in phase I/IIa trials, employs I.D. injection followed by electroporation to deliver a personalized DNA vaccine and led to significant neoantigen-specific CD4 and CD8 T cell responses in 86.4% of patients [101].

Numerous alternative methods also exist to target APCs, particularly DCs, to increase immunogenicity of DNA vaccines. For instance, DNA vaccines may encode the target antigen to heat shock protein 70 (HSP70) which can bind to DC surface receptors for receptor-mediated endocytosis and cross presentation [257]. For example, a naked DNA vaccine encoding HPV-16 E7 linked to HSP70 (pNGVL4a-Sig/E7 (detox)/HSP70 DNA vaccine) has been tested in the phase I/II clinical trial (NCT00121173) for patients with HPV16 + cervical precancerous lesions [258]. Fms-like tyrosine kinase 3 ligand (FLT3L) which can bind the DC FLT3 receptor to promote their proliferation and differentiation, has also been included in a DNA vaccine to enhance immune activation [259]. A phase II clinical trial (NCT02139267) tested GX-188E, a DNA vaccine for patients with HPV + cervical precancerous lesions encoding FLT3L together with HPV16 and HPV18 E6 and E7 genes [260]. Another method to enhance DC presentation is through DNA vaccine encoding the target antigen with calreticulin (CRT), which signals DCs to phagocytose the antigen and process it for MHC presentation [261]. For instance, a phase I clinical trial tested pNGVL4a-CRT/E7(Detox), an HPV DNA vaccine for HPV + head and neck cancer (NCT01493154).

Furthermore, addressing the rapid degradation of naked DNA by nucleases is important to increase their efficacy. By protecting the DNA from degradation by encapsulation with synthetic carriers, delivery across the cell membrane can be improved. For instance, a phase II/III clinical trial (NCT00264732) tested Amolimogene (ZYC101a), a plasmid DNA encoding HPV-16 and HPV-18 E6 and E7 epitopes encapsulated in poly-lactide co-glycolide microparticles, in patients with HPV + cervical precancerous lesions [262, 263]. Incorporating a nuclear localization signal (NLS) can also enhance DNA vaccine entry into the nucleus via nuclear pore complexes (NPCs) [264–266].

New forms of DNA vectors have gained interest recently, including minicircle DNA (mcDNA), nanoplasmid DNA, and doggybone DNA (dbDNA). Compared to traditional plasmid DNA (pDNA), these new forms eliminate the prokaryotic sequences which have been shown to produce inflammatory reactions, and they lack the genes for antibiotic resistance which could increase the incidence of antibiotic-resistant infections [267]. Their resulting smaller backbone size also provides a further benefit in enhancing transfection efficiency [268, 269]. mcDNA, or “minicircles” were first introduced in 1997 [270]. They are derived from traditional pDNA, which are then introduced into bacteria and excised by a recombinase enzyme to remove the bacterial backbone. This leaves only the “minicircle” containing the gene of interest and necessary regulatory elements [271, 272]. Despite its improvements over pDNA, the cost to manufacture mcDNA is high due to the complicated production process, and the short bacterial sequence still remains post-recombination. Nanoplasmid DNA, generated in the early 200 s, improves upon this by using an “antibiotic-free selection system” using an antisense RNA (RNA-OUT) to repress expression of a counter-selectable marker (sacB), which enables for plasmid selection without the need for antibiotic resistance markers [273, 274]. This method still retains minimal bacterial backbone, but has shown promising transfection efficiency up to ten times that of pDNA [269, 275]. More recently in 2017, dbDNA was able to eliminate the need for bacterial sequences through a fully in vitro process involving enzymes (Phi29 DNA polymerase and a protelomerase) to generate linear, covalently closed DNA [276]. This novel method is thus capable of rapid and large-scale production of the minimal construct, making it suitable for advancing gene therapies.

## Limitations in cancer vaccine efficacy and potential solutions

Even with the translation of a potent cancer vaccine from bench to bedside, there are still limitations that may hinder its efficacy. These obstacles can be distinguished from factors affecting vaccine-induced T cells and the tumor microenvironment itself.

First, cancer cells may opt for low MHC class I expression, which enhances their resistance against cytotoxic T cell activity. This downregulation can result from various defects in the MHC class I pathway, including mutational, transcriptional, translational, post-transcriptional, post-translational, and epigenetic alterations [277]. Therefore, a primary strategy is to upregulate MHC class I expression using cytokines (TNF, IL-1, type I and II interferons), STING agonists, or TLR agonists [278]. For instance, Propper et al. demonstrated that low-dose IFN-γ administration can increase MHC class I and II expression in metastatic melanoma [279]. Another strategy is chemoradiation, but this method can increase both MHC class I expression and tumor immunosuppression [280–282]. Therefore, further studies are required to determine the optimal dose of chemoradiation to minimize the immunosuppressive side effects. A recent approach involves using small-molecule inhibitors to block key proteins that dysregulate the global regulatory network for antigen presentation [283]. However, this strategy would entail a genome-wide screening first to identify novel regulatory proteins that contribute to the subversion of MHC molecules [283].

Another major obstacle is the inherent immunosuppression of the tumor ecosystem, which constantly remodels tumor cells, blood vessels, and cancer-associated fibroblasts to favor tumor growth and survival by curbing immune cell function [284, 285]. First, this characteristic can manifest as a physical barrier blocking out immune cells. A well-studied example is the desmoplastic nature of PDAC, which physically impedes T cell migration into and within the TME [286, 287]. There are several strategies to overcome this physical barrier, which includes blocking the TGF $\beta$, Wnt-$\beta$-catenin, and Hippo signaling pathways [288–290]. Second, transmigration of vaccine-induced T cells can be hindered by poor vascularization, which is often observed in pancreatic ductal adenocarcinoma too [291]. Consequently, this may also contribute to low intratumoral chemokine levels. It has been shown that low CCL4, CCL5, and CCL20 can impair DC entry into the tumor microenvironment for cross-presentation [292, 293]. General strategies include the use of chemo or radiotherapy to promote vascular leakage. Olive et al. reported a unique strategy to increase vascularization by disrupting the hedgehog signaling to reduce tumor-associated stromal fibroblasts [294].

In addition, many immunosuppressive cells are present in the tumor environment, causing impaired effector functions. Immunosuppressive players include regulatory T cells, phenotypic-M2 macrophages, cancer-associated fibroblasts, and myeloid-derived suppressor cells [295, 296]. T cell effector functions can be compromised when PD-1 on T cells contact with PD-L1 ligands [297, 298]. In addition, cytokines—such as IL-10 and TGF$\beta$—and metabolites—such as IDO—can suppress effector function and proliferation of vaccine-induced CD4 + and CD8 + T cells [299–301]. On the macroscopic scale, physical conditions such as hypoxia, low extracellular pH, and high interstitial fluid pressure can all contribute to impairment of these tumor-infiltrating T cells [302]. It is currently difficult to devise a strategy against these immunosuppressive factors because the intratumoral landscape is highly complex and dynamic in nature. Some efforts to reverse this immunosuppression include the use of ICI, hypoxia-directed cytotoxic drugs, and blockage of suppressive metabolite production using small-molecule inhibitors [303–305].

High tumor burden and hypoxia often lead to T cell exhaustion, which could limit vaccine effectiveness as it leads to diminished T cell activity and proliferation, as well as increased expression of inhibitory receptors and T cell death [306]. T cell dysfunction and exhaustion are driven by persistent antigen exposure leading to sustained TCR stimulation and subsequent metabolic dysregulation and upregulation of inhibitory receptors, as well as metabolic stress induced by the hypoxic environment. Combating potential T cell exhaustion in vaccine-induced T cells may involve concurrent immune checkpoint blockade, inhibiting DNA methylation enzymes known to induce exhaustion-specific DNA methylation patterns in T cells (such as DNMT3A, DNMT1, and DNMT3B), or reducing overactive TCR signaling by blocking downstream kinases [307, 308]. In addition, targeting metabolic pathways (such as inhibiting excessive glycolysis or enhancing fatty acid oxidation to support memory T cell development), or combination with adoptive T cell therapy may increase the efficacy of neoantigen vaccines.

Finally, in advanced cancers, efficacy of neoantigen vaccines may not be as effective due to challenges of tumor size and metastasis. While small, pre-metastatic tumors may be effectively treated with neoantigen vaccines, metastatic cancers may undergo epitope editing and clonal selection which can reduce vaccine efficacy, and the differing metastatic TMEs may create an additional challenge [309]. In such cases, combination therapies of surgery, chemo or radiotherapy, or other immunotherapies such as adoptive T cell transfer together with neoantigen vaccination may be necessary for clinical benefit [307].

## Clinical considerations and future directions

Personalized cancer vaccines have been utilized in two general clinical settings: one as an adjuvant to standard therapies for surgically resectable, relatively early-stage cancers, and the second targeting advanced, metastatic cancers as a maintenance treatment. In adjuvant settings, the clinical trials administer the cancer vaccines after the patients have undergone cancer resection. The vaccine is often administered with follow-up chemotherapy or checkpoint inhibitor therapy to eliminate residual cancer cells and prevent micrometastases, with the primary endpoint typically being RFS**.** Examples of this strategy include Moderna's mRNA-4157 for surgically resected melanoma (NCT03897881, NCT05933577) and surgically resected NSCLC (NCT06077760), Transgene's TG4050 for surgically resected head and neck squamous cell carcinoma of the head and neck (HNSCC) (NCT04183166), and BioNTech and Genentech's autogene cevumeran for surgically resected pancreatic ductal adenocarcinoma (PDAC) (NCT04161755, NCT05968326) [1, 5, 6].On the other hand, for trials targeting metastatic cancers, the goal is to maintain disease control. Therefore, the primary endpoints are typically progression-free survival (PFS) or molecular readouts representing disease control. Examples include BioNTech and Genentech's autogene cevumeran for metastatic, unresectable melanoma (NCT03815058), Gritstone's GRANITE (GRT-C901/GRT-R902) for metastatic microsatellite stable colorectal cancer (MSS-CRC) (NCT05141721), and Evaxion's EVX-01 for metastatic, unresectable melanoma (NCT05309421).

There are pros and cons to testing these personalized cancer vaccines in these two demographics. Targeting surgically resectable cancer could reach a larger patient population, as earlier stage cancers are more common. However, these trials are financially demanding due to the long-term follow-up required to determine recurrence rates. For example, in Moderna’s mRNA-4157 phase II trial for melanoma (KEYNOTE-942/NCT03897881), RFS was monitored for up to 5 years. Conversely, targeting advanced disease is traditionally preferred for new cancer therapeutics due to enhanced patient recruitment and quicker outcomes. However, cancer vaccines tested in advanced disease patients may not necessarily demonstrate effectiveness, as their efficacy depends on a healthy immune system, which may be compromised in late-stage patients.

The current momentum seems to favor the use of personalized cancer vaccines as an adjuvant treatment in surgically resectable cancers. As of April 2024, vaccine candidates for surgically resectable cancers have reported promising results. The mRNA-4157 in combination with pembrolizumab demonstrated longer recurrence-free survival and less recurrence and death rate than pembrolizumab alone in a phase IIb study for surgically resected melanoma [5]. Transgene also reported no recurrence in the vaccine group in a phase I study of TG4050 for surgically resected head and neck cancer (NCT04183166). Conversely, Gritstone reported suboptimal interim analysis of phase II trial using GRT-C901/GRT-R902 for metastatic MSS-CRC, where circulating tumor DNA (ctDNA) reduction was used as the primary endpoint [310].

The promising results of vaccines in surgically resectable cancer may be due to the effectiveness of T cells in eliminating cancer cells before an immunosuppressive TME is established. Additionally, in patients with advanced cancer, the impaired host immunity may hamper robust vaccination effects. Therefore, the needle-to-needle time between biopsy for personalized cancer vaccine development and vaccination in late-stage cancers becomes an important consideration. One strategy is to combine the use of shared antigen vaccines and personalized vaccines. In this case, following biopsy, the patient would first receive a dose of an off-the-shelf shared antigen (TSA/TAA) vaccine that is available for common HLA types before later receiving doses of personalized cancer vaccine.

**Fig. 4:**
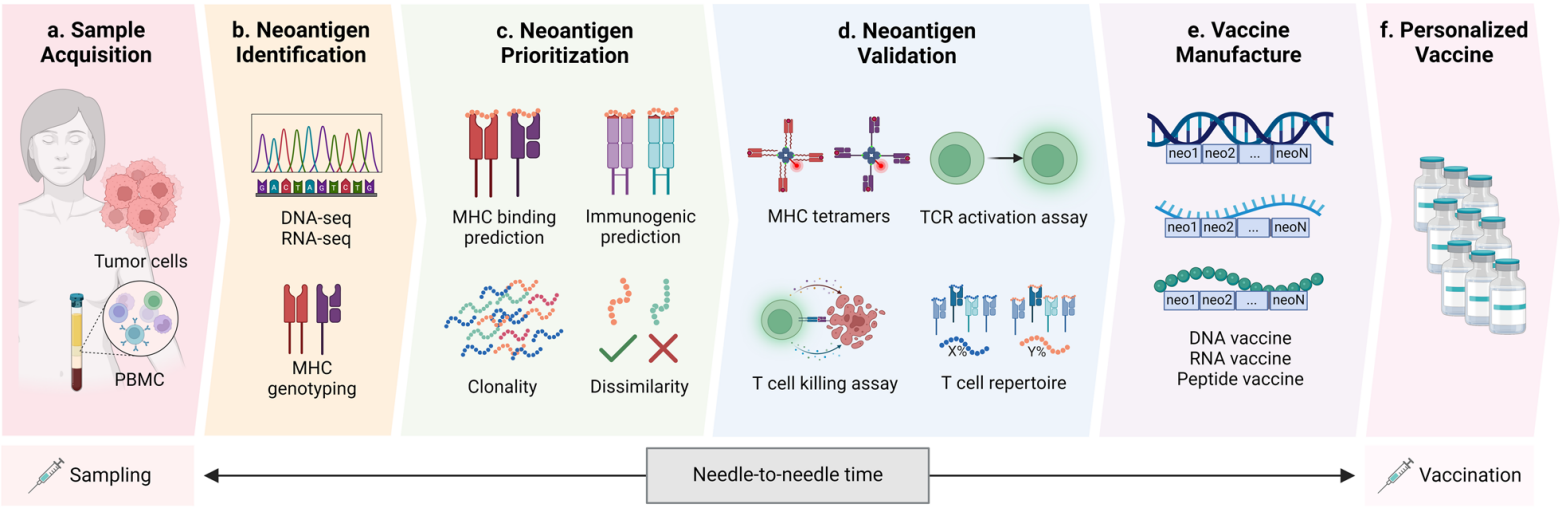
Pipeline for personalized cancer vaccine development. **a** Sample acquisition. Samples are acquired from patients’ tumor biopsies, with PBMCs or matched normal tissues as germline controls. **b** Neoantigen identification. Potential neoantigens are then identified from these samples. For mutation-dependent neoantigen identification, variant callers can be used to identify genetic variants in DNA- or RNA-seq data. For mutation-independent neoantigen identification, RiboSeq and/or immunopeptidomics can identify cryptic neoantigens generated from alterations in transcription or translation. Sequencing data is further used to determine the individual’s MHC type (MHC genotyping). **c** Neoantigen prioritization. Assessment of neoantigen immunogenicity and safety is done through neoantigen prioritization, including MHC binding prediction to identify and prioritize neoantigens with high binding affinity to a patient’s unique MHC molecule, immunogenic prediction to determine binding efficiency between the TCR and MHC-peptide complex for T cell activation, determination of neoantigen clonality to effectively target the tumor, and neoantigen dissimilarity from self-peptide to prevent autoimmune response. **d** Neoantigen validation. Upon identifying potential neoantigen candidates, functional assays are crucial for their validation. To identify neoantigen-specific T cells, MHC tetramers may be synthesized and loaded with the neoantigenic peptides to bind these T cells for quantification by flow cytometry or cell sorting. Functional response of T cells to the neoantigens may be measured by TCR activation assays or T cell killing assays. Finally, the TCR repertoire may be analyzed via sequencing methods to assess the diversity and clonality of the neoantigen-specific T cell response. **e** Vaccine manufacture. The target neoantigens can then be synthesized into cancer vaccines, most commonly peptide, DNA, and RNA vaccines. **f** Personalized vaccine. The final personalized vaccine may include adjuvants or utilize a particular delivery platform (such as nanoparticles) to enhance delivery efficiency and immunogenicity. The needle-to-needle time at the bottom of the figure indicates the time from acquisition of the tumor sample to the generation of the personalized vaccine—this process must be done in a timely manner in order to ensure therapeutic effect for the patient

In the future, administering cancer vaccines in patients with earlier-stage/pre-cancer lesions can position this treatment as a preventive cancer vaccine for high-risk populations. The preventive vaccination would include frequently occurring cancer neoantigens, which can be identified by analyzing a growing number of patients over time or through analysis of databases such as The Cancer Genome Atlas (TCGA) and Genotype-Tissue Expression project (GTEx). Figure 4 provides a summary of the developmental pipeline of personalized cancer vaccines, from sample acquisition to vaccine production.

## Conclusions

The success of cancer vaccines hinges on several factors, including neoantigen quality, vaccine platform, vaccination obstacles, and host immune system. High quality neoantigens are typically tumor-specific, predominantly clonal, and immunogenic. Although it remains challenging to predict an antigen’s TCR binding affinity and immunogenicity, more data and better algorithms are driving this field forward. The advent of newer and flexible vaccine platforms is changing the vaccine field. However, we should take into consideration that different vaccine platforms may induce different patterns of CD4 or CD8 T cell responses [311]. Furthermore, we must overcome several obstacles to effective cancer vaccination, including the immunosuppressive features of the TME, T cell exhaustion, and challenges of tumor size and metastasis. Finally, a functional host immune system is crucial to translate a vaccine’s immunogenicity to its clinical efficacy. Vaccines should not only activate T cells but also generate memory responses to ensure long-term control. To address this, there have been several clinical trials combining cancer vaccines with ICI therapies or chemotherapies or using cancer vaccines as an adjuvant therapy post-surgery in early-stage cancers [312]. Moreover, recent advancements in vaccine platforms and computational tools have led to promising results in clinical trials. We anticipate that therapeutic cancer vaccines will play a crucial role in cancer immunotherapy in the foreseeable future.

## Supplementary Information

Supplementary material 1: Table 1. mRNA Vaccines Targeting Tumor-Associated Antigens. Table 2. DNA Vaccines Targeting Tumor-Associated Antigens. Table 3. mRNA Vaccines Targeting Viral Tumor-Specific Antigens. Table 4. DNA Vaccines Targeting Viral Tumor-Specific Antigens. Table 5. mRNA Vaccines Targeting Personalized Neoantigens. Table 6. DNA Vaccines Targeting Personalized Neoantigens. Table 7. mRNA-loaded Dendritic Cell Vaccine.

Supplementary material 2: Table 1. Methods for MHC I and II Genotyping. Table 2. Methods for MHC Binding Prediction. Table 3. Methods for TCR Binding Prediction.

## Data Availability

All data relevant to this review are included in the article or uploaded as supplementary information. Data and materials are available upon reasonable request.

## Abbreviations

AML: Acute myeloid leukemia
APC: Antigen-presenting cells
BCG: Bacillus Calmette-Guerin
CDR: Complementarity-determining regions
CDS: Coding sequence
ChAdV: Chimpanzee adenovirus vector
COVID-19: Coronavirus disease-2019
CPTAC: Clinical Proteomic Tumor Analysis Consortium
CRC: Colorectal cancer
CTA: Cancer-testis antigen
ctDNA: Circulating tumor DNA
CTL: Cytotoxic lymphocytes
CTNNB1: Catenin beta 1
DC: Dendritic cells
DMFS: Distant metastasis-free survival
DNA: Deoxyribonucleic acid
DNP: Dinitrophenol
EBV: Epstein-Barr virus
EGFR: Epidermal growth factor receptor
ELISpot: Enzyme-linked immunosorbent spot assay
FACS: Fluorescence-activated cell sorting
FDA: Food and Drug Administration
FFPE: Formalin-fixed and paraffin-embedded
GAd: Gorilla adenovirus
GM-CSF: Granulocyte–macrophage colony-stimulating factor
GTEx: Genotype-Tissue Expression project
HBV: Hepatitis B virus
HCC: Hepatocellular carcinoma
HER: Human epidermal growth factor receptor
HLA: Human leukocyte antigen
HNSCC: Head and neck squamous cell carcinoma
HPV: Human papillomavirus
hTERT: Human telomerase reverse transcriptase
ICI: Immune checkpoint inhibitors
IDO: Indoleamine 2,3-dioxygenase
IFN-γ: Interferon gamma
IGFBP2: Insulin-like growth factor binding protein 2
IL: Interleukin
KRAS: Kirsten rat sarcoma viral oncogene homologue
LNP: Lipid nanoparticles
LPA: Lipid particle aggregates
MAGE: Melanoma antigen gene
MFE: Minimum free energy
MHC class I: Major histocompatibility complex class I
MHC class II: Major histocompatibility complex class II
mRNA: Messenger ribonucleic acid
MSS: Microsatellite stable
NGS: Next-generation sequencing
NSCLC: Non-small cell lung cancer
NY-ESO-1: New York esophageal squamous cell carcinoma 1
PAP: Prostatic acid phosphatase
PBMC: Peripheral blood mononuclear cells
PD-L1: Programmed death-ligand 1
PDAC: Pancreatic ductal adenocarcinoma
pMHC: MHC-peptide complex
PRAME: Preferentially expressed antigens in melanoma
PSA: Prostate-specific antigen
PSCA: Prostate stem cell antigen
PSMA: Prostate-specific membrane antigen
RBP: Ribosome binding proteins
RFS: Recurrence-free survival
RiboSeq: Ribosome profiling
RNA-seq: RNA sequencing
SLP: Synthetic long peptides
SNV: Single nucleotide variant
STING: Stimulator of interferon genes
TAA: Tumor-associated antigen
TCGA: The Cancer Genome Atlas
TCR: T cell receptor
TIL: Tumor infiltrating lymphocytes
TLR: Toll-like receptor
TMB: Tumor mutational burden
TME: Tumor microenvironment
TNF: Tumor necrosis factor
TP53: Tumor protein p53
TSA: Tumor-specific antigen
WES: Whole exome sequencing
WGS: Whole genome sequencing
WT1: Wilms tumor 1
