# Supplementary material for: Molecular targets and strategies in the development of nucleic acid cancer vaccines: from shared to personalized antigens
Source: J Biomed Sci. 2024 Oct 9;31:94. doi: 10.1186/s12929-024-01082-x (PMC11463125; doi:10.1186/s12929-024-01082-x)
Supplement: Supplementary file 1 — Supplementary material 1: Table 1. mRNA Vaccines Targeting Tumor-Associated Antigens. Table 2. DNA Vaccines Targeting Tumor-Associated Antigens. Table 3. mRNA Vaccines Targeting Viral Tumor-Specific Antigens. Table 4. DNA Vaccines Targeting Viral Tumor-Specific Antigens. Table 5. mRNA Vaccines Targeting Personalized Neoantigens. Table 6. DNA Vaccines Targeting Personalized Neoantigens. Table 7. mRNA-loaded Dendritic Cell Vaccine. [file 12929_2024_1082_MOESM1_ESM.docx]

**Table 1. mRNA Vaccines Targeting Tumor-Associated Antigens**

| Vaccine Target | Vaccine Name | Cancer Type | Delivery Method | Combination | Phase | NCT Number |
| --- | --- | --- | --- | --- | --- | --- |
| CD40L, CD70, acTLR4 | TriMix mRNA | Breast Cancer | mRNA | - | I | NCT03788083 |
| CLDN6 | BNT142 | CLDN6+ Solid Tumors | mRNA-LPX | - | I/II | NCT05262530 |
| Melan-A, MAGE-A1, MAGE-A3, survivin, gp100, tyrosinase | - | Melanoma | mRNA-protamine complex | - | I/II | NCT00204607 |
| NY-ESO-1, MAGE-A3, tyrosinase, TPTE | BNT111 (Lipo-MERIT) | Melanoma | mRNA-LPX | cemiplimab | I/II | NCT04526899, NCT02410733 |
| MAGE-A3, MAGE-C2, tyrosinase, gp100 | ECI-006 | Melanoma | mRNA | - | I | NCT03394937 |
| NY-ESO-1,MAGE-C1, MAGE-C2, survivin, 5T4, | CV9201 | NSCLC | mRNA-protamine complex | - | I/II | NCT00923312 |
| NY-ESO-1, MAGE-C1, MAGE-C2, survivin, 5T4,MUC1 | CV9202 | NSCLC | mRNA-protamine complex | durvalumab and tremelimumab | I/II | NCT03164772 |
| Undisclosed tumor-associated antigens | BNT-116 | NSCLC | mRNA-LPX | cemiplimab | II | NCT05557591, NCT05142189 |
| PAP, kallikrein-2, kallikrein-3, HOXB13, NK3 homeobox 1 | BNT112 (PRO-MERIT) | Prostate cancer | mRNA-LPX | cemiplimab | I/II | NCT04382898 |
| PSA, PSCA, PSMA, STEAP1, | CV9103 | Prostate Cancer | mRNA-protamine complex | - | I/II | NCT00831467 |
| NY-ESO-1, MAGE-C1, MAGE-C2, 5 T4, survivin, MUC1 | CV9202 (RNActive) | Prostate Cancer and NSCLC | mRNA-protamine complex | durvalumab and tremelimumab | I | NCT03164772 |
| PD-L1, IDO1 | mRNA-4359 | Solid tumors | mRNA | pembrolizumab | I/II | NCT05533697 |

**5T4, Trophoblast Glycoprotein; CD40L, CD40 Ligand; CD70, Cluster of Differentiation 70; CLDN6, Claudin 6; gp100, Glycoprotein 100; HOXB13, Homeobox B13; IDO1, Indoleamine 2,3-Dioxygenase 1; mRNA, Messenger Ribonucleic Acid; MAGE, Melanoma Antigen Gene; MUC1, Mucin 1; NCT, National Clinical Trial; NK3, Natural Killer-3; NY-ESO-1, New York Esophageal Squamous Cell Carcinoma-1; PAP, Prostatic Acid Phosphatase; PD-L1, Programmed Death-Ligand 1; PSA, Prostate-Specific Antigen; PSCA, Prostate Stem Cell Antigen; PSMA, Prostate-Specific Membrane Antigen; STEAP1, Six Transmembrane Epithelial Antigen of the Prostate 1; TLR, Toll-Like Receptor; TPTE, Transmembrane Phosphatase with Tensin Homology.*

**Table 2 DNA Vaccines Targeting Tumor-Associated Antigens**

| Vaccine Target | Vaccine Name | Cancer Type | Delivery Method | Combination | Phase | NCT Number |
| --- | --- | --- | --- | --- | --- | --- |
| NY-ESO-1 | pPJV7611 | NY-ESO-1+ cancer: NSCLC, Prostate, Bladder, Esophageal, Sarcoma | DNA plasmid via PMED | - | I | NCT00199849 |
| MAGE-A1, MAGE-A3, TH, Phox2B, Survivin, PRAME; fused with PVXCP | - | Neuroblastoma | DNA-polyethylenimine conjugate via attenuated Salmonella enterica (oral administration) | - | I | NCT04049864 |
| hTERT | INVAC1 (IVS-2001) | Solid tumors | DNA plasmid via I.D. injection and electroporation | - | I | NCT02301754, NCT04515043 |
| hTERT | TRT-001 (INO-1400 or INO-1401) | Solid tumors | DNA plasmid via I.M. injection and electroporation | INO-9012 (encoding IL-12) | I | NCT02960594 |
| hTERT, WT1, PSMA | INO-5401 | Glioblastoma, and urothelial carcinoma | DNA plasmid via I.M. injection and electroporation | *In GBM:* INO-9012, Cemiplimab, Temozolomide, radiation  *In UCa:* INO-9012, Atezolizumab | I/II | NCT03491683, NCT03502785 |
| HER2 | AST201 or AST301 | Breast, gastric, and ovarian Cancer | DNA plasmid via I.D. injection | *In breast:* rhuGM-CSF, Pembrolizumab or Capecitabine  *In gastric:* rhuGM-CSF  *In ovarian:* rhuGM-CSF, Paclitaxel or Carboplatin | II | NCT05163223, NCT05771584, NCT05794659 |
| HER2, IGFBP2, IGF1R | WOKVAC (pUMVC3-IGFBP2-HER2-IGF1R) | Breast cancer | DNA plasmid via I.D. injection | Paclitaxel, Trastuzumab, Pertuzumab | II | NCT04329065 |
| HER3 | pING-hHER3FL | HER3+ cancer | DNA plasmid via I.M. injection | - | I | NCT03832855 |
| PSA, TRICOM (CD80, LFA-3, ICAM-1) | [PROSTVAC-VF](https://ascopubs.org/doi/10.1200/JCO.2021.39.6_suppl.TPS192) | Prostate cancer | DNA via fowlpox virus | Nivolumab and Ipilimumab | I | [NCT03532217](https://www.tandfonline.com/doi/full/10.1517/13543780902997928) |

**CD80, Cluster of Differentiation 80; DNA, Deoxyribonucleic Acid; GM-CSF, Granulocyte-Macrophage Colony-Stimulating Factor; HER2, Human Epidermal Growth Factor Receptor 2; hTERT, Human Telomerase Reverse Transcriptase; ICAM-1, Intercellular Adhesion Molecule 1; IGF1R, Insulin-like Growth Factor 1 Receptor; IGFBP2, Insulin-like Growth Factor Binding Protein 2; IL-12, Interleukin 12; LFA-3, Lymphocyte Function-associated Antigen 3; MAGE, Melanoma Antigen Gene; NCT, National Clinical Trial; NY-ESO-1, New York Esophageal Squamous Cell Carcinoma-1; PRAME, Preferentially Expressed Antigen in Melanoma; PSMA, Prostate-Specific Membrane Antigen; WT1, Wilms Tumor 1.*

**Table 3. mRNA Vaccines Targeting Viral Tumor-Specific Antigens**

| Vaccine Target | Vaccine Name | Cancer Type | Delivery Method | Combination | Phase | NCT Number |
| --- | --- | --- | --- | --- | --- | --- |
| HPV16 E6/E7 | BNT113 | HNSCC | mRNA-LPX | pembrolizumab | I/II | NCT04534205, NCT03418480 |
| EBV-associated antigens | WGC-043 | EBV-positive advanced malignant tumors | mRNA | - | I | NCT05714748 |
| HBV-associated antigens | - | Hepatocellular carcinoma | mRNA | - | I | NCT05738447 |

**EBV, Epstein-Barr Virus; HNSCC, Head and Neck Squamous Cell Carcinoma; HPV, Human Papillomavirus; mRNA, Messenger Ribonucleic Acid; NCT, National Clinical Trial.*

**Table 4. DNA Vaccines Targeting Viral Tumor-Specific Antigens**

| Vaccine Target | Vaccine Name | Cancer Type | Platform | Combination | Phase | NCT Number |
| --- | --- | --- | --- | --- | --- | --- |
| HPV16 E6/E7 | VB10.16 | HPV+ cervical or head-neck squamous cell carcinoma | DNA plasmid via I.M. injection | *In HPV+ CC:* Atezolizumab  *In HPV+ HNSCC:* Pembrolizumab | II (HPV+ CC)  I/II (HPV+ HNSCC) | [NCT06099418](https://classic.clinicaltrials.gov/ct2/show/NCT06099418), NCT06016920 |
| HPV16 E6, HPV18 E6/E7 | PVX7 (pBI-11 & TA-HPV) | HPV+ oropharyngeal cancer | DNA plasmid via I.M. injection | Pembrolizumab | II | NCT05799144 |
| HPV16 E6/E7, HPV18 E6/E7 | VGX-3100 | HPV+ cervical cancer | DNA plasmid via I.M. and electroporation | - | III | NCT03185013, NCT03721978 |
| HPV16 E6/E7, HPV18 E6/E7 | GX-188E | HPV+ cervical cancer | DNA plasmid via I.M. injection and electroporation | Pembrolizumab | I/II | NCT03444376 |
| HPV16 E6/E7-CRT fusion protein | pNGVL4aCRTE6E7L2 | HPV+ cervical neoplasia | DNA plasmid via I.M. injection | TA-CIN protein vaccine boost | I | NCT03913117 |
| HPV16 E7 | pBI-11 & TA-HPV  pNGVL4a-Sig/E7 (Detox)/HSP70 | HPV+ cervical neoplasia | DNA plasmid via I.M. injection | Imiquimod | I  I/II | NCT00788164, NCT00121173 |

**CC, Cervical Cancer; DNA, Deoxyribonucleic Acid; HPV, Human Papillomavirus; HSP70, Heat Shock Protein 70; I.M., Intramuscular; NCT, National Clinical Trial.*

**Table 5. mRNA Vaccines Targeting Personalized Neoantigens**

| Vaccine Target | Vaccine Name | Cancer Type | Delivery Method | Combination | Phase | NCT Number |
| --- | --- | --- | --- | --- | --- | --- |
| Patient-specific neoantigens | IVAC_W_bre1_uID/IVAC_M_uID | Breast cancer | mRNA-LPX | - | I | NCT02316457 |
| Patient-specific neoantigens | GRT-C901 + GRT-R902 | CRC | mRNA-LNP | atezolizumab, ipilimumab, fluoropyrimidine + leucovorin, or bevacizumab | II/III | NCT05141721 |
| Patient-specific neoantigens | BNT122/Autogene cevumeran/ RO7198457 | CRC | mRNA-LPX | - | II | NCT04486378 |
| Patient-specific neoantigens | PGV002 | Esophageal cancer, gastric cancer, liver cancer | mRNA | anti-PD-1/L1 | - | NCT05192460 |
| Patient-specific neoantigens | - | Esophageal cancer, NSCLC | mRNA | - | - | NCT03908671 |
| Patient-specific neoantigens | - | Gastric cancer | mRNA | anti-PD-1/L1 | - | NCT05227378 |
| Patient-specific neoantigens, pp65-LAMP | - | Glioblastoma | mRNA-LNP | - | I | NCT04573140 |
| Patient-specific neoantigens | ABOR2014/IPM511 | Liver cancer | mRNA | - | I | NCT05981066 |
| Patient-specific neoantigens | BNT121 (IVAC-MUTANOME) | Melanoma | mRNA | RBL001/RBL002 | I | NCT02035956 |
| Patient-specific neoantigens | mRNA-4157/V940 | Melanoma | mRNA-LNP | pembrolizumab | III | NCT05933577 |
| Patient-specific neoantigens | RBL001/RBL002 | Melanoma | mRNA | - | I | NCT01684241 |
| Patient-specific neoantigens | BNT122/Autogene cevumeran/ RO7198457 | Melanoma | mRNA-LPX | pembrolizumab | II | NCT03815058 |
| Patient-specific neoantigens | mRNA-4157/V940 | Melanoma | mRNA-LNP | pembrolizumab | II | NCT03897881 |
| Patient-specific neoantigens | mRNA-4157/V940 | NSCLC | mRNA-LNP | pembrolizumab | III | NCT06077760 |
| Patient-specific neoantigens | BNT122 (RO7198457) | Pancreatic cancer | mRNA | atezolizumab | I | NCT04161755 |
| Patient-specific neoantigens | XH001 | Solid tumors | mRNA | sintilimab | - | NCT05940181 |
| Patient-specific neoantigens | HRXG-K-1939 | Solid tumors | mRNA | adebrelimab | I | NCT05942378 |
| Patient-specific neoantigens | BNT122/Autogene cevumeran/ RO7198457 | Solid tumors | mRNA-LPX | atezolizumab | I | NCT03289962 |
| Patient-specific neoantigens | mRNA-4157/V940 | Solid tumors | mRNA-LNP | pembrolizumab | I | NCT03313778 |
| Patient-specific neoantigens | mRNA-0217/S001 | Solid tumors | mRNA-LNP | pembrolizumab | I | NCT05916248, NCT05916261 |
| Patient-specific neoantigens | SW1115C3 | Solid tumors | mRNA | - | I | NCT05198752 |
| Patient-specific neoantigens | JCXH-212 | Solid tumors | mRNA | toripalimab | I | NCT05579275 |
| Patient-specific neoantigens | mRNA-4157/V940 | Squamous cell carcinoma | mRNA-LNP | pembrolizumab | II/III | NCT06295809 |

** anti-PD-1/L1, Anti-Programmed Cell Death Protein 1 (PD-1) or its Ligand 1; CRC, Colorectal Cancer; LNP, Lipid Nanoparticle; LPX, Lipid Particle; mRNA, Messenger Ribonucleic Acid; NCT, National Clinical Trial; NSCLC, Non-Small Cell Lung Cancer.*

**Table 6. DNA Vaccines Targeting Personalized Neoantigens**

| Vaccine Target | Vaccine Name | Cancer Type | Delivery Method | Combination | Phase | NCT Number |
| --- | --- | --- | --- | --- | --- | --- |
| Up to 30 antigens (27 TSA and 3 patient-specific) | GNOS-PV01 | Unmethylated glioblastoma | DNA plasmid via I.M. injection and electroporation | INO-9012 (plasmid encoding IL-12) | I | NCT04015700 |
| Up to 40 patient-specific neoantigens | GNOS-PV02 | Hepatocellular carcinoma | DNA plasmid via I.D. injection and electroporation | INO-9012 & pembrolizumab | I/II | NCT04251117 |
| Patient-specific neoantigens | Neoantigen DNA vaccine | Prostate cancer | DNA plasmid via I.M. injection and electroporation | Nivolumab (anti-PD-1), Ipilimumab (anti-CTLA-4), PROSTVAC (PSA vaccine) | I | NCT03532217 |
| Patient-specific neoantigens | Personalized neoantigen DNA vaccine | Brain tumors | DNA plasmid via I.M. injection and electroporation | - | I | NCT03988283 |
| Patient-specific neoantigens | Neoantigen DNA vaccine | SCLC | DNA plasmid via I.M. injection and electroporation | Durvalumab, (and Carboplatin, Etoposide) | II | NCT04397003 |
| 4-20 patient-specific neoantigens | Personalized polyepitope DNA vaccine | TNBC | DNA plasmid via I.M. injection and electroporation | - | I | NCT02348320 |
| Patient-specific neoantigens | Personalized Neoantigen DNA vaccine | Glioblastoma | DNA plasmid via I.M. injection and electroporation | Retifanlimab | I | NCT05743595 |
| Up to 40 patient-specific neoantigens | VB10.NEO | Solid tumors | DNA plasmid via I.M. injection | - Atezolizumab  - CPI (anti-PD-1 or anti-PD-L1), Bempegaldesleukin | I  I/II | NCT05018273, NCT03548467 |
| Up to 60 patient-specific neoantigens | NOUS-PEV | NSCLC and melanoma | Gorilla adenovirus prime, modified vaccinia Ankara boost | Pembrolizumab | I | NCT04990479 |
| Up to 30 patient-specific neoantigens | TG4050 | Head and neck cancer and ovarian cancer | Modified vaccinia Ankara | - | I | NCT03839524, NCT04183166 |
| Up to 20 patient-specific neoantigens | GRT-C901 + GRT-R902; GRANITE-001 (ZVexNeo) | NSCLC, CRC, gastroesophageal, and urothelial cancer | Chimpanzee adenovirus prime, self-amplifying RNA boost-LNP | Fluoropyrimidine, bevacizumab | II/III | NCT05141721, NCT03639714 |

**CRC, Colorectal Cancer; DNA, Deoxyribonucleic Acid; IL-12, Interleukin-12; I.D., Intradermal; I.M., Intramuscular; LNP, Lipid Nanoparticle; NCT, National Clinical Trial; NSCLC, Non-Small Cell Lung Cancer; RNA, Ribonucleic Acid; SCLC, Small Cell Lung Cancer; TNBC, Triple-Negative Breast Cancer.*

**Table 7. mRNA-loaded Dendritic Cell Vaccine**

| **Vaccine Target** | **Vaccine Name** | **Cancer Type** | **Delivery Method** | **Combination** | **Phase** | **NCT Number** |
| --- | --- | --- | --- | --- | --- | --- |
| hTERT, LAMP-1 | GRNVAC1 | AML | mRNA-loaded dendritic cell | - | II | NCT00510133 |
| WT1, PRAME, CMVpp65 | - | AML | mRNA-loaded dendritic cell | - | I/II | NCT01734304 |
| WT1 | - | AML | mRNA-loaded dendritic cell | - | I | NCT00834002 |
| WT1 | CPX-351 | AML | mRNA-loaded dendritic cell | low-dose chemotherapy | II | NCT01686334 |
| WT1, hTERT, survivin | - | AML | mRNA-loaded dendritic cell | - | I | NCT05000801 |
| hTERT, survivin, p53 | - | Breast Cancer | mRNA-loaded dendritic cell | cyclophosphamide | I | NCT00978913 |
| CEA | - | CRC | mRNA-loaded dendritic cell | - | I/II | NCT00228189 |
| WT1 | - | Glioblastoma | mRNA-loaded dendritic cell | temozolomide and radiation | I/II | NCT02649582 |
| WT1 | - | High-grade Glioma, Diffuse Intrinsic Pontine Glioma | mRNA-loaded dendritic cell | chemoradiation | I/II | NCT04911621 |
| WT1 | - | Malignant Pleural Mesothelioma | mRNA-loaded dendritic cell | conventional chemotherapy | I/II | NCT02649829 |
| MAGE-A3, MAGE-C2, tyrosinase, gp100 | TriMix-DC-MEL | Melanoma | mRNA-loaded dendritic cell | - | I | NCT01066390 |
| MAGE-A3, MAGE-C2, tyrosinase, gp100 | TriMix-DC-MEL | Melanoma | mRNA-loaded dendritic cell | ipilimumab | II | NCT01302496 |
| Tyrosinase, gp100 | - | Melanoma | mRNA-loaded dendritic cell | cisplatin | II | NCT02285413 |
| Tyrosinase, gp100 | TriMix-DC and TLR-DC | Melanoma | mRNA-loaded dendritic cell | - | I/II | NCT01530698, NCT00243529 |
| Tyrosinase, gp100 | TriMix-DC | Melanoma | mRNA-loaded dendritic cell | - | I/II | NCT00243529 |
| Trp2 | - | Melanoma | mRNA-loaded langerhans-type dendritic cell | - | I | NCT01456104 |
| Melan-A, Mage-A1, Mage-A3, survivin, GP100, tyrosinase | - | Melanoma | mRNA-loaded dendritic cell | GM-CSF | I/II | NCT00204516 |
| CT7, MAGE-A3, WT1 | - | Multiple Myeloma | mRNA-loaded langerhans-type dendritic cell | - | I | NCT01995708 |
| PSA, PAP, survivin, hTERT | - | Prostate cancer | mRNA-loaded dendritic cell | docetaxel | II | NCT01446731 |
| Prostate cancer tissue, hTERT, survivin | - | Prostate Cancer | mRNA-loaded dendritic cell | - | I/II | NCT01197625 |
| CMV pp65-LAMP | - | Glioblastoma | mRNA-loaded dendritic cell | basiliximab | I | NCT00626483 |
| CMV pp65-LAMP | - | Glioblastoma | mRNA-loaded dendritic cell | autologous lymphocyte transfer | I | NCT00639639 |
| CMV pp65-LAMP | - | Glioblastoma | mRNA-loaded dendritic cell | basiliximab + temozolomide | II | NCT02366728 |
| CMV pp65-LAMP | - | Glioblastoma | mRNA-loaded dendritic cell | GM-CSF + temozolomide | I | NCT03615404 |
| CMV pp65-LAMP | - | Glioblastoma | mRNA-loaded dendritic cell | temozolomide + varlilumab | II | NCT03688178 |
| CMV pp65-LAMP | - | Glioblastoma | mRNA-loaded dendritic cell | GM-CSF | II | NCT02465268 |
| CMV pp65-LAMP | - | Glioma, Astrocytoma, Glioblastoma | mRNA-loaded dendritic cell | surgical resection + nivolumab | I | NCT02529072 |
| Patient-specific neoantigens | PerCellVac2 | Glioblastoma | mRNA-loaded dendritic cell | - | I | NCT02808416, NCT02808364 |
| Patient-specific neoantigens | - | Glioblastoma | mRNA-loaded dendritic cell | temozolomide | I | NCT02709616 |
| Patient-specific neoantigens | TTRNA-DC | Glioma | mRNA-loaded dendritic cell | GM-CSF, cyclophosphamide, fludarabine, ex-vivo tumor-reactive lymphocytes | I | NCT03396575 |
| Patient-specific neoantigens | TTRNA-DC | Medulloblastoma, neuroectodermal tumors | mRNA-loaded dendritic cell | ex-vivo tumor reactive lymphocytes | I/II | NCT01326104 |
| Patient-specific neoantigens | - | Melanoma | mRNA-loaded dendritic cell | - | III | NCT01983748 |
| Patient-specific neoantigens | - | Melanoma | mRNA-loaded dendritic cell | IL-2 | I/II | NCT01278940 |
| Patient-specific neoantigens | - | Pancreatic cancer | mRNA-loaded dendritic cell | standard chemotherapy | I | NCT04157127 |
| Patient-specific neoantigens, hTERT, survivin | - | Prostate cancer | mRNA-loaded dendritic cell | - | I/II | NCT01197625 |
| Patient-specific neoantigens | AGS-003 | Renal cell carcinoma | mRNA-loaded dendritic cell | sunitinib | II | NCT00678119 |
| Patient-specific neoantigens | MB-002 | Renal cell carcinoma | mRNA-loaded dendritic cell | CD40L | I/II | NCT00087984 |

**AML, Acute Myeloid Leukemia; CT7, Cancer/Testis Antigen 7; CEA, Carcinoembryonic Antigen; CD40L, CD40 Ligand; CRC, Colorectal Cancer; CMVpp65, Cytomegalovirus phosphoprotein 65; DC, Dendritic Cell; gp100, Glycoprotein 100; GM-CSF, Granulocyte-Macrophage Colony-Stimulating Factor; hTERT, human Telomerase Reverse Transcriptase; IL-2, Interleukin-2; LAMP-1, Lysosome-Associated Membrane Protein 1; MAGE, Melanoma-Associated Antigen; NCT, National Clinical Trial; PSA, Prostate-Specific Antigen; PAP, Prostatic Acid Phosphatase; PRAME, Preferentially Expressed Antigen in Melanoma; RNA, Ribonucleic Acid; p53, Tumor Protein 53; Trp2, Tyrosinase-Related Protein 2; WT1, Wilms Tumor 1.*
