# Supplementary material for: Molecular targets and strategies in the development of nucleic acid cancer vaccines: from shared to personalized antigens
Source: J Biomed Sci. 2024 Oct 9;31:94. doi: 10.1186/s12929-024-01082-x (PMC11463125; doi:10.1186/s12929-024-01082-x)
Supplement: Supplementary file 2 — Supplementary material 2: Table 1. Methods for MHC I and II Genotyping. Table 2. Methods for MHC Binding Prediction. Table 3. Methods for TCR Binding Prediction. [file 12929_2024_1082_MOESM2_ESM.docx]

**Supplementary Table 1. Methods for MHC I and II Genotyping**

| Tool | Input | Description | Website Details |
| --- | --- | --- | --- |
| Optitype | WES/WGS/RNA-seq | MHC I | <https://academic.oup.com/bioinformatics/article/30/23/3310/206910> |
| Polysolver | WES | MHC I | <https://www.nature.com/articles/nbt.3344> |
| HLAMatchmaker | Amino acid sequences | MHC I | <https://www.sciencedirect.com/science/article/pii/S0198885902003828> |
| PHLAT | WES/WGS/RNA-seq | MHC I + II | <https://link.springer.com/article/10.1186/1471-2164-15-325> |
| seq2HLA | RNA-seq | MHC I + II | <https://link.springer.com/article/10.1186/gm403> |
| HLAreporter | WES | MHC I + II | <https://link.springer.com/article/10.1186/s13073-015-0145-3> |
| HLAforest | RNA-seq | MHC I + II | <https://journals.plos.org/plosone/article?id=10.1371/journal.pone.0067885> |
| HLAminer | WES/WGS/RNA-seq/Long Reads | MHC I + II | <https://link.springer.com/article/10.1186/gm396> |
| xHLA | WGS/WES | MHC I + II | <https://www.pnas.org/doi/abs/10.1073/pnas.1707945114> |
| ArcasHLA | RNA-seq | MHC I + II | <https://academic.oup.com/bioinformatics/article/36/1/33/5512361> |
| Athlates | WES | MHC I + II | <https://academic.oup.com/nar/article/41/14/e142/1750166> |
| Immuannot | WGS | MHC I + II | <https://www.biorxiv.org/content/10.1101/2024.01.20.576452v1.abstract> |
| SoapHLA | Target capture sequencing / WGS | MHC I + II | <https://www.ncbi.nlm.nih.gov/pmc/articles/PMC3722289/> |
| Kourami | WGS/WES | MHC I + II | <https://genomebiology.biomedcentral.com/articles/10.1186/s13059-018-1388-2> |
| HLA-HD | WES/WGS/RNA-seq/Long reads | MHC I + II | <https://onlinelibrary.wiley.com/doi/10.1002/humu.23230> |
| HLA*LA (formerly HLA*PRG) | WGS/WES | MHC I + II | <https://academic.oup.com/bioinformatics/article/35/21/4394/5426702> |
| HLAProfiler | RNA-seq | MHC I + II | <https://genomemedicine.biomedcentral.com/articles/10.1186/s13073-017-0473-6> |

**HLA, Human Leukocyte Antigen; MHC, Major Histocompatibility Complex; RNA-seq, RNA sequencing; WES, Whole Exome Sequencing; WGS, Whole Genome Sequencing.*

**Supplementary Table 2. Methods for MHC Binding Prediction**

| Tool | Year | MHC | Method/Algorithm | Training data | Website Details |
| --- | --- | --- | --- | --- | --- |
| SYFPEITHI | 1999 | Class I + II | PSSM | MHC binding affinity | <https://pubmed.ncbi.nlm.nih.gov/10602881/> |
| TEPITOPE | 1999 | Class II | LR | MHC binding affinity | <https://pubmed.ncbi.nlm.nih.gov/10385319/> |
| RANKPEP | 2002 | Class I | PSSM | MHC binding affinity | <https://pubmed.ncbi.nlm.nih.gov/12175724/> |
| SMM | 2005 | Class I | PSSM | MHC binding affinity | <https://bmcbioinformatics.biomedcentral.com/articles/10.1186/1471-2105-6-132> |
| SMM_align | 2007 | Class II | LR | MHC binding affinity | <https://bmcbioinformatics.biomedcentral.com/articles/10.1186/1471-2105-8-238> |
| Combinatorial library | 2008 | Class II | LR | MHC binding affinity | <https://www.ncbi.nlm.nih.gov/pmc/articles/PMC2248166/> |
| PickPocket | 2009 | Class I | PSSM | MHC binding affinity | <https://www.ncbi.nlm.nih.gov/pmc/articles/PMC2732311/> |
| SMMPMBEC | 2009 | Class I | PSSM | MHC binding affinity | <https://bmcbioinformatics.biomedcentral.com/articles/10.1186/1471-2105-10-394> |
| NetMHCII | 2009 | Class II | NN | MHC binding affinity | <https://bmcbioinformatics.biomedcentral.com/articles/10.1186/1471-2105-10-296> |
| IEDB-AR-Consensus | 2012 | Class I + II | Concensus | - | <https://www.ncbi.nlm.nih.gov/pmc/articles/PMC6602498/> |
| NetMHCcons | 2012 | Class I | Concensus | MHC binding affinity | <https://link.springer.com/article/10.1007/s00251-011-0579-8> |
| TEPITOPEpan | 2012 | Class II | LR | MHC binding affinity | <https://journals.plos.org/plosone/article?id=10.1371/journal.pone.0030483> |
| OWA-PSSM | 2013 | Class II | PSSM | MHC binding affinity | <https://www.ncbi.nlm.nih.gov/pmc/articles/PMC3908610/> |
| NetMHCstab | 2014 | Class I | NN | MHC binding specificity | <https://pubmed.ncbi.nlm.nih.gov/23927693/> |
| NetMHC4 | 2015 | Class I | NN | MHC binding affinity | <https://academic.oup.com/bioinformatics/article/32/4/511/1744469> |
| NetMHCstabpan | 2016 | Class I | NN | MHC binding specificity | <https://services.healthtech.dtu.dk/services/NetMHCstabpan-1.0/> |
| PSSMHCpan | 2017 | Class I | PSSM | MHC binding affinity | <https://pubmed.ncbi.nlm.nih.gov/28327987/> |
| MixMHCpred | 2017 | Class I | PSSM | MS elute peptides | <https://journals.plos.org/ploscompbiol/article?id=10.1371/journal.pcbi.1005725> |
| NetMHCpan4.0 | 2017 | Class I | NN | MHC binding affinity + MS eluted peptides | <https://www.ncbi.nlm.nih.gov/pmc/articles/PMC5679736/> |
| MHCnuggets | 2017 | Class I + II | NN (LSTM) | MS eluted peptides + MHC binding affinity | <https://pubmed.ncbi.nlm.nih.gov/31871119/> |
| ConvMHC | 2017 | Class I | NN (CNN) | MHC binding affinity | <https://pubmed.ncbi.nlm.nih.gov/29281985/> |
| HLA-CNN | 2017 | Class I | NN (CNN) | MHC binding affinity | <https://pubmed.ncbi.nlm.nih.gov/28444127/> |
| EDGE | 2018 | Class I | NN | MS elute peptides | <https://www.nature.com/articles/nbt.4313> |
| NetMHCIIpan | 2018 | Class II | NN | MHC binding affinity | <https://europepmc.org/article/med/29315598> |
| HLAthena | 2019 | Class I | NN | MS elute peptides | <https://www.nature.com/articles/s41587-019-0322-9> |
| Neonmhc2 | 2019 | Class II | CNN | MS elute peptides | <https://www.sciencedirect.com/science/article/pii/S1074761319303632> |
| MARIA | 2019 | Class II | RNN | MS elute peptides + antigen gene expression + protease cleavage signatures | <https://www.nature.com/articles/s41587-019-0280-2> |
| DeepHLApan | 2019 | Class I | RNN | Peptide Binding and Immunogenicity | <https://www.frontiersin.org/journals/immunology/articles/10.3389/fimmu.2019.02559/full> |
| MixMHC2pred | 2019 | Class II | LR | MS elute peptides (peptidome) | <https://www.nature.com/articles/s41587-019-0289-6> |
| MHCflurry 2.0 | 2020 | Class I | NN | MHC binding affinity | <https://www.sciencedirect.com/science/article/pii/S2405471220302398> |
| BigMHC | 2023 | Class I | NN ensemble | MS elute peptides + antigen-specific immune response | <https://www.nature.com/articles/s42256-023-00694-6> |

** CNN, convolutional neural network; LR, linear regression; MHC, Major Histocompatibility Complex; MS, Mass Spectrometry; NN, neural network; LSTM, long short-term memory; PSSM, position-specific scoring matrix; RNN, recurrent neural network*

**Supplementary Table 3. Methods for TCR Binding Prediction**

| Tool | Year | Training set | Method | Website Details |
| --- | --- | --- | --- | --- |
| tcrdist | 2017 | CDR3α + CDR3β | distance-based | <https://www.nature.com/articles/nature22383> |
| TCRex | 2018 | CDR3β | random forests | <https://link.springer.com/article/10.1007/s00251-017-1023-5> |
| NetTCR | 2018 | CDR3β | CNN | <https://www.biorxiv.org/content/10.1101/433706v1> |
| MAIT Match | 2019 | CDR3α | similarity-scoring approach | <https://www.nature.com/articles/s42003-019-0442-2> |
| ImRex | 2020 | CDR3α + CDR3β | CNN | <https://academic.oup.com/bib/article/22/4/bbaa318/6042663?login=false> |
| SETE | 2020 | CDR3β | PCA + decision tree | <https://www.sciencedirect.com/science/article/abs/pii/S1476927120303194?via%3Dihub> |
| ERGO | 2020 | CDR3β | NLP | <https://www.frontiersin.org/journals/immunology/articles/10.3389/fimmu.2020.01803/full> |
| TcellMatch | 2020 | CDR3α + CDR3β | NN | <https://www.embopress.org/doi/full/10.15252/msb.20199416> |
| TCRMatch | 2020 | CDR3β | similarity-scoring approach | <https://www.frontiersin.org/journals/immunology/articles/10.3389/fimmu.2021.640725/full> |
| NetTCR2.0 | 2021 | CDR3α + CDR3β | CNN | <https://www.nature.com/articles/s42003-021-02610-3> |
| ERGO-II | 2021 | CDR3α + CDR3β | RNN (LSTM encoder) | <https://www.frontiersin.org/journals/immunology/articles/10.3389/fimmu.2021.664514/full> |
| TITAN | 2021 | CDR3β | CNN + Attention | <https://academic.oup.com/bioinformatics/article/37/Supplement_1/i237/6319659?login=false> |
| pMTnet | 2021 | CDR3β | LSTM | <https://www.nature.com/articles/s42256-021-00383-2> |
| TCRGP | 2021 | CDR1/2/3α + CDR1/2/3β | Gaussian process classification | <https://pubmed.ncbi.nlm.nih.gov/33764977/> |
| TCRAI | 2021 | CDR3α + CDR3β | CNN | <https://www.science.org/doi/10.1126/sciadv.abf5835> |
| ATM-TCR | 2022 | CDR3β | Multi-Head Self-Attention Model | <https://www.frontiersin.org/journals/immunology/articles/10.3389/fimmu.2022.893247/full> |
| NetTCR2.1 | 2022 | CDR3α + CDR3β | CNN | <https://www.frontiersin.org/journals/immunology/articles/10.3389/fimmu.2022.1055151/full> |
| MixTCRpred | 2024 | CDR1/2/3α + CDR1/2/3β | NN | <https://www.nature.com/articles/s41467-024-47461-8> |
| NetTCR2.2 | 2024 | CDR1/2/3α + CDR1/2/3β | CNN | <https://elifesciences.org/reviewed-preprints/93934> |

********CDR, Complementarity Determining Region; CNN, Convolutional Neural Network; LSTM, Long Short-Term Memory; NLP, Natural Language Processing; NN, Neural Network; PCA, Principal Component Analysis; RNN, Recurrent Neural Network; TCR, T-cell Receptor.*
